# Evaluation of the Embrittlement in Reactor Pressure-Vessel Steels Using a Hybrid Nondestructive Electromagnetic Testing and Evaluation Approach

**DOI:** 10.3390/ma17051106

**Published:** 2024-02-28

**Authors:** Gábor Vértesy, Madalina Rabung, Antal Gasparics, Inge Uytdenhouwen, James Griffin, Daniel Algernon, Sonja Grönroos, Jari Rinta-Aho

**Affiliations:** 1 HUN-REN Centre for Energy Research, 1121 Budapest, Hungary; 2 Fraunhofer Institute for Nondestructive Testing (IZFP), 66123 Saarbrücken, Germany; madalina.rabung@izfp.fraunhofer.de; 3 SCK CEN Belgian Nuclear Research Centre, 2400 Mol, Belgium; inge.uytdenhouwen@sckcen.be; 4 Centre for Manufacturing and Materials, Coventry University, Coventry CV1 5FB, UK; ac0393@coventry.ac.uk; 5 SVTI Swiss Association for Technical Inspections, 8304 Wallisellen, Switzerland; daniel.algernon@svti.ch; 6 VTT Technical Research Centre of Finland Ltd., P.O. Box 1000, FI-02044 Espoo, Finland; smgronroos@gmail.com (S.G.); jari.rinta-aho@helsinki.fi (J.R.-A.)

**Keywords:** reactor pressure vessel, neutron-irradiation-generated embrittlement, electromagnetic nondestructive evaluation

## Abstract

The nondestructive determination of the neutron-irradiation-induced embrittlement of nuclear reactor pressure-vessel steel is a very important and recent problem. Within the scope of the so-called NOMAD project funded by the Euratom research and training program, novel nondestructive electromagnetic testing and evaluation (NDE) methods were applied to the inspection of irradiated reactor pressure-vessel steel. In this review, the most important results of this project are summarized. Different methods were used and compared with each other. The measurement results were compared with the destructively determined ductile-to-brittle transition temperature (DBTT) values. Three magnetic methods, 3MA (micromagnetic, multiparameter, microstructure and stress analysis), MAT (magnetic adaptive testing), and Barkhausen noise technique (MBN), were found to be the most promising techniques. The results of these methods were in good agreement with each other. A good correlation was found between the magnetic parameters and the DBTT values. The basic idea of the NOMAD project is to use a multi-method/multi-parameter approach and to focus on the synergies that allow us to recognize the side effects, therefore suppressing them at the same time. Different types of machine-learning (ML) algorithms were tested in a competitive manner, and their performances were evaluated. The important outcome of the ML technique is that not only one but several different ML techniques could reach the required precision and reliability, i.e., keeping the DBTT prediction error lower than a ±25 °C threshold, which was previously not possible for any of the NDE methods as single entities. A calibration/training procedure was carried out on the merged outcome of the testing methods with excellent results to predict the transition temperature, yield strength, and mechanical hardness for all investigated materials. Our results, achieved within the NOMAD project, can be useful for the future potential introduction of this (and, in general, any) nondestructive evolution method.

## 1. Introduction

In the majority of industrial countries, nuclear power plants (NPPs) are used worldwide to generate electricity. There are two reasons for the current use of nuclear power: increased world prices of fossil fuels and the fear of climate change due to increased CO_2_ emissions during combustion. Today, 353 reactors in the world are older than 25 years, among which 154 are above 40 years. The long-term operation (LTO) of existing NPPs has already been accepted in many countries as a strategic objective to ensure the adequate supply of electricity over the coming decades [1,2]. The public and energy experts may disagree as to whether or not nuclear reactors are indispensable as an energy source for today’s human society, but both supporters and opponents of nuclear energy agree that the safety of reactors must be maintained at the highest possible levels.

The design lifetimes are affected by operating conditions, such as neutron exposure (fluence), and also by the magnitude and number of temperature and/or pressure cycles [3,4]. The two basic regulatory approaches are a license renewal and periodic safety reviews, which are required for the authorization of the long-term operation of NPPs [1]. An evaluation of these parameters makes possible an estimation of the operational NPPs’ lifetime [3]. 

One of the most important and irreplaceable parts that limits the lifetime of NPPs is the steel reactor pressure vessel (RPV), which encloses the extremely radioactive part of the whole system. An RPV must be tough, solid, strong, and absolutely reliable. It should never be cracked or leak or in any way allow the radioactive contents to escape from inside. However, it is well-known that the mechanical properties of the RPV wall are modified during its operation [5]. The ageing of the structure of the RPV steel near the reactor core is generated caused by long-term and high-energy neutron irradiation and also by thermal effects. These effects change the microstructure of the steel and make the RPV material become increasingly more brittle, making it more susceptible to unwanted cracks. 

The evaluation of these parameters allows for an estimation of the operational lifetime of NPPs. Currently, destructive tests are performed on surveillance samples within the scope of periodic safety reviews (PSRs) in order to assess the material degradation induced by the neutron irradiation in the RPVs. The Charpy impact test, which is performed on surveillance specimens, is presently the traditional and highly reliable way of performing RPV toughness inspections. Surveillance specimens are standard tensile and ISO-V Charpy specimens of exactly the same RPV steels and their welds [6]. These specimens experience exactly the same history as does the RPV steel of the vessel. It is considered that their physical conditions vary during the entire lifetime of the reactor in the same way as that of the vessel. Their physical condition also testifies to the “near future” condition of the vessel material. 

The neutron-irradiation-induced embrittlement is usually described by the ductile-to-brittle transition temperature (DBTT). To obtain a single DBTT value, several specimens must be tested. For assessing structural material information over a long period, a lot of Charpy samples are needed. Meanwhile, tensile specimens tested under a quasistatic loading rate are used to determine the yield strength, tensile strength, uniform elongation, total elongation, and reduction in diameter.

Due to possible material heterogeneities such as macro-segregated regions, hydrogen flakes, and the inclusion areas in such large components, the surveillance specimens might not necessarily represent the whole vessel material. Furthermore, destructive methods do not allow for the characterization of the progress of material properties of the same specimen when successively damaged. They are not applicable to the actual component, and, finally, the amount of Charpy surveillance samples is limited for performing monitoring over an elongated period. 

It is evident that a nondestructive technique of RPV steel material testing can avoid many of the aforementioned problems. It could be a nondestructive inspection of an alternative series of surveillance specimens, which would be measured outside of the pressure vessel, returned back into the vessel after each investigation, and then measured again at the next periodic inspection phase. Alternatively, special inspection methods and devices employing nondestructive techniques could be utilized, enabling an inside inspection of the vessel with the aid of specially equipped electrical leads. Yet a non-invasive evaluation of the vessel material could be performed, focusing on carefully chosen external areas of the vessel whenever feasible.

However, unlike mechanical tests, nondestructive tests do not directly measure the material property. Currently, no single nondestructive test has replaced existing testing methods such as the destructive Charpy test.

A project (“NOMAD”), oriented towards the development of novel electromagnetic nondestructive methods for the inspection of operation-induced material degradation in nuclear power plants, received funding from the Euratom research and training program. The purpose of this review paper is to give a comprehensive survey of the results achieved in the project. In the project, a combination of several nondestructive methods was used to examine how accurately and reliably the degradation of cladded RPV material could be determined, especially when compared to sample-based destructive testing. Charpy samples, cladded and non-cladded blocks of various RPV steels, along with different levels of irradiation states, were provided. Different nondestructive evaluation techniques were adapted and applied to measure the irradiated and non-irradiated samples. The applied NDE methods were magnetic, electrical, thermoelectric, and ultrasonic methods. Destructive reference tests for the determination of the DBTT and other material characteristics were carried out as well. The irradiated samples were handled and measured in hot cells. The measured data were both evaluated and correlated with the destructively determined DBTT values using machine-learning methods. In this way, a predictive model was generated which is able to nondestructively determine the DBTT for various RPV steel types by intelligently combining the information of several nondestructive testing methods. The outcome of the data-driven approach was finally validated. 

The validation was established by a detailed evaluation of the functionality of the NOMAD tool. This was conducted in the context of the regression approach as well as being complemented by a classification approach. Furthermore, the evaluation provided insight into the relevance of the different measurement features. Based on these achievements, recommendations were made under the assumption of a continuation of the development toward a higher TRL.

A short description of the physical background of the radiation embrittlement of RPV steel is devoted in Section 2. The state of the art regarding nondestructive investigation before the NOMAD project is summarized in Section 3. Electromagnetic nondestructive tests of various irradiated RPV steels are summarized in Section 4. The purpose of the NOMAD project is described in Section 5. The sample preparation and description of destructive tests are described in Section 6 and Section 7. The results of the measurement, performed in the project, on both Charpy and block specimens are summarized and, in addition, different methods are compared with each other in Section 8. Section 9 is devoted to studying the evaluation of the multi-output-parameter (i.e., combined) NDE technique with the help of advanced classification methods, particularly machine-learning (ML) techniques and their applicability. Section 10 is the discussion and interpretation of the project, while the summary and conclusions (Section 11) of the whole project and recommendations are given for future research and the application of the investigated techniques. 

The different methods applied in the project are described in detail in Appendix A, while in Appendix B, the interpretation of the scatter of the measured points is analyzed. 

## 2. The Radiation Embrittlement

The main ageing process of reactor pressure vessels is the irradiation embrittlement of the metallic structural materials. A large number of experimental and theoretical studies have been published on the influence of radiation embrittlement. Several reviews have been published about this phenomenon [7,8,9,10,11,12,13,14,15,16,17,18].

Radiation embrittlement is caused by high-energy fast neutron radiation (over 0.1 MeV), and high-energy gamma radiation. Embrittlement refers to a decrease in the fracture toughness of reactor vessel materials and affects the vessel materials in the vicinity of the reactor fuel, referred to as the vessel’s “beltline”. It is one of the most significant lifespan degradation phenomena of both the pressurized and boiling water reactors. Consequently, the monitoring of this ageing process is of primary interest to nuclear power plant operators and regulatory bodies.

**Figure 1:**
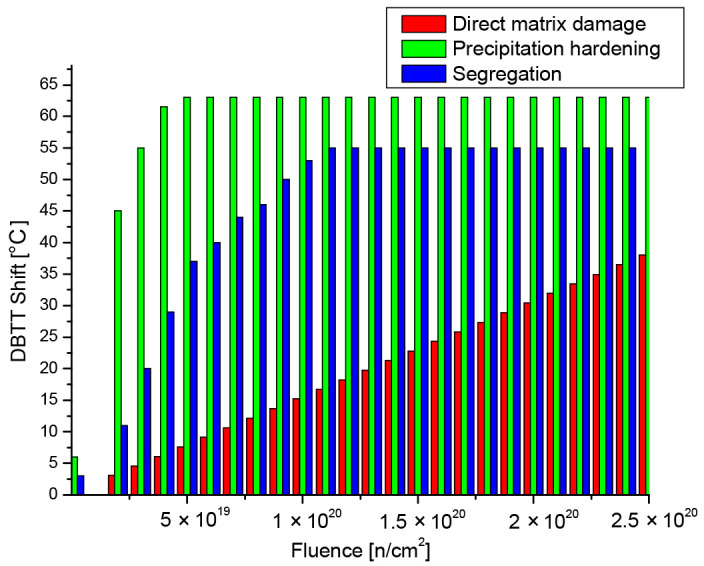
The characteristics of radiation embrittlement processes: the change in the transition temperature (DBBT) as a function of the E > 0.1 MeV neutron fluence.

The radiation-induced embrittlement is a complex process, and it comprises several different processes. The most notable ones are shown in Figure 1, which are discussed in the literature [13]. This complexity in combination with the material inhomogeneity makes the testing and evaluation challenging especially in a nondestructive way. Furthermore, these processes progress with different characteristics as the material is exposed to the neutron irradiation, the key processes are as follows:

- Direct matrix damage, caused by the increase in the dislocation density due to neutron bombardment;
- Precipitation hardening of the matrix, here the most important element is Cu, obviously others like Ni, Mn, Si, etc. also have a diminishing contribution;
- Segregation, (P is a recognized segregating element) and if P covers the grain boundary even only in one-atom thickness it can cause non-hardening embrittlement.

The neutron-irradiation-generated direct matrix damage can be characterized by a simple root square dependence on fluence for a given material and at a given temperature. Since the atoms’ mobility increases at higher irradiation temperatures, the rate of damage is considered to be decreasing thanks to diffusion. During direct matrix damage formation, Cu, as well as other elements, is known to lead to precipitation mechanisms of nano-precipitates, also inducing matrix hardening and embrittlement. This mechanism continues until saturation, which depends on the available amount of precipitants. As mentioned, Cu concentration has a particular role here. In addition, other elements, like P, can segregate in the grains and at the grain boundary (through diffusion processes), also in combination with matrix damage or attracted into the Cu-type precipitates. Definitely, the diffusion of segregates also takes part in this mechanism, but this aspect is rather difficult to characterize. P segregation in the grain boundaries generally does not cause hardening.

There is a so-called master curve approach for assessing the fracture toughness of an irradiated reactor pressure vessel (RPV) steel. This approach is accepted worldwide nowadays. Since it is a direct measurement approach, it prevails against any correlative or indirect methods applied previously for assessing the irradiated RPV integrity. The usability of master curve testing has been illustrated by Wallin [19]. Moreover, this approach has been applied utilizing ASTM Standard Test Method E 1921 [20] in the USA [21].

Furthermore, several other effects have to be considered. Thermal ageing and thermal annealing are accelerated by irradiation (irradiation speeds up the diffusion processes even at relatively low temperatures). These processes are time-dependent; consequently, the irradiation flux rate may also affect the rate of the embrittlement. Through the cross section of an RPV wall (especially in the case of forgings), the fracture toughness properties are changing and the rate of thermal ageing is different [22,23,24]. Significant differences can be found in thermal expansion coefficients with respect to pressure vessel base metals, which can cause a stress peak [25]. This is the so-called pressurized thermal shock and it is a potential risk to interfacial crack initiation and propagation. Safety analysis of this phenomenon has lately become a subject of interest for operators of NPPs [26]. 

There is a literature review available that presents the current understanding of the mechanisms behind radiation-induced embrittlement in low alloy reactor pressure-vessel steels and irradiation-assisted stress corrosion cracking in the core internal structure of stainless steels [27]. 

## 3. NDT Methods—State of the Art

As outlined in the previous sections, the mechanical properties of the reactor pressure vessel wall are modified during its operation. As a result, the regular inspection of NPPs is an extremely important task. The DBTT, measured by destructive Charpy tests, is the standardized parameter in the nuclear industry which characterizes the embrittlement. A significant drawback of this method is that many samples are necessary to be used for this inspection. Additionally, the measurement error is high. Because of these arguments, different nondestructive methods have also been recommended and applied for monitoring the degradation of nuclear pressure-vessel steel material. The life prediction and NDE of material properties in the power plant industry are given in [28].

However, as already mentioned in the introduction, nondestructive tests cannot directly measure the material property that needs to be determined—unlike destructive methods. They can measure quantitative values of other physical properties (like different electromagnetic parameters), which are influenced by similar material properties, such as by the presence and quality of structural material defects for instance, and therefore correlate with the mechanical properties. The reliable way to obtain quantitative information on a destructive-test-based required property, such as the DBTT, when using nondestructive measurements is through establishing a credible one-to-one correlation between the destructively and nondestructively measured properties. This requires a series of comparative experimental investigations. All nondestructive tests must be rigorously correlated with relevant destructive tests through multiple series of checked and re-checked measurements before reliable application. This lack of correlation is the primary reason that no nondestructive test has been able to replace destructive tests, or even be applied as an auxiliary test, which could build a strong foundation for correlation verification. Nevertheless, a number of potential nondestructive tests have been recommended and tested, and the mosaic of the requested correlations is presently being built up. 

These tests could be conducted with the aid of armored electrical leads through special bushing/containment within the vessel. Furthermore—at least in principle—an appropriate test could be a nondestructive quality inspection of the vessel material itself, at different well-selected positions, even, from outside of the vessel. Several indirect physical methods (e.g., magnetic, ultrasound, acoustic, X-rays, etc.) of nondestructive testing exist, and they can be principally applied for such inspections.

A number of nondestructive methods have recently been suggested for studying the neutron-irradiation-generated embrittlement of RPV steel material. The paper [29] gives a summary of electromagnetic techniques used for the nondestructive testing (NDT) of degradation of nuclear reactor components. However, since neutron irradiation makes the investigated material radioactive and this causes significant difficulty in their measurement, simulation techniques, such as ion irradiation, thermal ageing, and cold working, can also be used to simulate irradiation damage [30,31,32]. 

### 3.1. Piezoelectric Ultrasound (Piezo-US)

The well-known ultrasonic method is widely applied in the inspection of NPPs [33,34,35]. Piezoelectric transducers use the so-called piezoelectric effect to excite ultrasonic waves. This effect is based on the occurrence of an electrical voltage when piezoelectric crystals are mechanically expanded and compressed (direct piezoelectric effect). Applying an electrical voltage to these crystals will, in turn, lead to their deformation proportional to the electric field (indirect piezoelectric effect). Thus, by using the piezoelectric effect, electrical energy is transformed into mechanical energy and vice versa. The indirect piezoelectric effect is used to transmit ultrasonic waves; the direct one is used to receive ultrasonic waves. As in this case the ultrasonic waves are generated in the transducers themselves, a coupling agent is needed to transfer these waves into the test object as well as to receive them.

The ultrasonic time-of-flight (TOF) method can be used as well, as the main measured quantity of the ultrasonic testing method. The TOF is defined as the time the ultrasonic wave requires for propagating from transmitter to receiver. For a given wave mode, microstructure changes and mechanical properties influence the effective ultrasound velocity and, therefore, they affect the TOF. The TOF is determined by fitting a peak of the received waveform with a parabolic function and determining the peak position analytically. The achievable accuracy lies in the single-digit nanosecond range [36,37].

### 3.2. Electrical Methods

#### 3.2.1. Thermoelectric Power Measuring Method (TEPMM)

Thermoelectricity is based on the fact that a thermal flux driven by a temperature gradient in an electrically conductive material is accompanied by an electric current. The voltage or thermoelectric power (TEP) created hereby is proportional to the temperature gradient. The material-dependent proportionality factor is called the Seebeck coefficient (SC). Today, thermoelectric devices used as temperature sensors (thermocouples), heat sources or sinks, and remote power generators are based on thermoelectric effects and are widely used. The change in the SC due to neutron irradiation and thermal and mechanical ageing has been observed experimentally. These effects are well-known as a cause of drift in thermocouples, which are widely used as temperature sensors [38,39,40].

#### 3.2.2. Direct Current-Reversal Potential Drop (DCRPD)

The four-point probe direct current-reversal potential drop (DCRPD) method is based on the measurement of the electrical resistivity of a metal. Material property changes in irradiated RPV can be used as indicators for the state of degradation. One physical effect that can be used for the detection of irradiated material degradation is the electrical resistivity. If the change in electrical resistivity is a well-defined function of the neutron fluence, and if the effect is large enough compared with that of the other influencing parameters, it can be used to monitor the material embrittlement. The development and testing is conducted to investigate the suitability of electrical resistivity measurement for the characterization of the irradiation embrittlement in irradiated pressure-vessel steel. Characterizing an irradiated RPV base metal, electrical resistivity requires the measurement of very low resistance changes with a high accuracy [41,42].

### 3.3. Magnetic Methods

The majority of nuclear reactors which are used presently are pressurized water reactors. The steel used in the reactor pressure vessel is ferromagnetic, allowing for effective inspection using magnetic methods. Magnetic nondestructive methods are an important part of all the possible techniques, especially because of their simplicity. Furthermore, it is known and understood that the magnetic and mechanical properties of ferromagnetic materials are very closely correlated: the regularity of the microstructure of ferromagnetic construction materials (e.g., of RPVs) and the density and quality of its defects have a significant influence on both the mechanical and magnetic properties of the material. The domain wall motion and dislocation movement are both influenced by the microstructure of the material. The correlation between mechanical and magnetic hardness in ferromagnetic materials is well-known and understood [43,44]. The practical applicability of magnetic methods for the quantitative indication of steels’ micro-structural modifications resulting in embrittlement have been proved by numerous successful measurements. 

In [45,46,47,48,49], overviews can be found about nondestructive magnetic methods. These papers provide a guideline to the literature of magnetic techniques for nondestructive material testing. Compared to other NDE techniques such as ultrasonics or eddy currents, the literature on magnetic methods is limited, yet one of the challenges is its widespread dispersion. The abovementioned reviews present a fairly comprehensive summary of the works on the known methods, such as the Barkhausen noise effect, magnetoacoustic emission, magnetic hysteresis method, residual field, and magnetically induced velocity change methods that can be used in practice.

The well-known magnetic Barkhausen noise technique (MBN) was originally developed for the inspection of surface defects, of residual stresses, and of microstructure changes [50]. There is a wide choice in the literature regarding how MBN can be applied for material characterization [51,52,53,54,55,56,57,58,59,60].

Magnetoacoustic emission, another magnetic method, can also be successfully used for the monitoring of residual stresses [61,62].

A nondestructive magnetic method was previously developed, which is called the 3MA approach (3MA = micromagnetic, multiparameter, microstructure and stress analysis), where this technique uses different methods [63,64,65]. 

The measurement of the magnetic hysteresis loops is also among the perspective candidates of magnetic NDE. The theory of ferromagnetic hysteresis is given in several papers, see [66] as an example. 

The magnetic hysteretic characterization of ferromagnetic materials with objectives towards the NDE of material degradation was discussed in [67].

In [31], cold rolling was applied to generate crystalline defects in RPV steel. Magnetic hysteresis measurement together with Vickers hardness and tensile properties measurements were performed. The variations in remanence, hysteresis loss, and coercivity were discussed in detail. It was found that the strength, hardness, and coercivity increased with increasing deformation. A good linear correlation between the increment of coercivity, hardness, and yield strength was found.

In traditional measurements of magnetic hysteresis, the major loop is measured, and several characteristic parameters, like remanence magnetization, coercive force, and maximal permeability, are determined from the major loop. However, in recently developed novel methods, a series of minor loops are measured instead of the major loop, and a lot of different parameters of minor hysteresis loops are used for material characterization. One of these novel methods is the magnetic minor loops power scaling laws (PSL) [31,68]. A similar method, called magnetic adaptive testing (MAT), also systematically measures the minor magnetic hysteresis loops [69,70,71]. As was demonstrated, all 3MA, PSL, and MAT methods are multi-parametric, powerful, and sensitive methods of magnetic inspection. In the case of the MAT method, systematic comparisons have been made between different magnetic nondestructive techniques: different methods—full hysteresis loop measurement, MAT, and MBN—were applied in a series of plastically deformed transformation-induced plasticity (TRIP) steel specimens, and the results of these methods were compared with each other [72]. In conclusion, a good correlation was found between magnetic parameters, measured by different techniques, which is a direct proof that all magnetic methods accurately characterize the material degradation.

MAT seems to be the most sensitive method among the other investigated ones. 

A good correlation was also found between the magnetic parameters and with the destructively measured Vickers hardness values. This fact makes possible the future potential use of magnetic methods in the inspection of RPVs’ structural integrity.

All of these parameters were determined without magnetic saturation of the investigated samples. This fact is very important in practical applications.

In the next section, it will be reviewed how these methods have been applied for the investigation of the material degradation caused by neutron irradiation.

The methods 3MA, MAT, and MBN were applied in the NOMAD project.

## 4. Magnetic Nondestructive Tests of Various Neutron-Irradiated RPV Steels—State of the Art

The practical applicability of magnetic methods has been proved recently by several successful measurements. These methods are suitable for the quantitative indication of micro-structural modifications of RPV steel causing steel embrittlement. Based on these results, the future potential application of magnetic methods seems to be promising in the inspection of RPVs’ structural integrity. The possibilities and difficulties of the NDE evaluation of irradiation degradation are analyzed in Ref. [73]. There is a comprehensive summary about the investigation of hardening in neutron-irradiated and thermally aged iron-copper alloys on the basis of mechanical and magnetic relaxation phenomena in Ref. [74]. In this subsection, the results which were achieved in the area of magnetic nondestructive tests of various irradiated RPVs are shown, with separated results for each of the applied methods. 

### 4.1. Major Hysteresis Loop Measurements

The modification of the magnetic hysteretic behavior generated by neutron irradiation was studied for different materials in [75]. Two parameters of the hysteresis loop (maximum relative differential permeability and peak intensity of interaction field) were determined as a function of neutron fluence. A decreasing trend (change up to 40%) was found in magnetic parameters during embrittlement, regardless of the origin of the embrittlement.

The influence of the neutron irradiation on the M-H magnetization curve of the RPV material was studied in [76]. The saturation and the residual magnetic induction and the initial magnetic susceptibility were determined. It was found that the clockwise variation in the magnetization of the hysteresis curves before irradiation was lower than after irradiation. Furthermore, the magnetization of the specimens before and after irradiation was not sensitive to temperature changes. The residual magnetization intensity was found to be linearly related to the irradiation fluences of less than 0.154 dpa. An exponential relationship of initial magnetic susceptibility with radiation fluence was found as well. 

The modification of the saturation magnetization of RPV steel, caused by neutron irradiation was studied in [77]. Magnetic and metallurgical properties were investigated by hysteresis loop and ferromagnetic resonance (FMR) techniques. The saturation magnetization of neutron-irradiated steel increased. To explain the cause of this increase, FMR experiments were also conducted and a large difference was experienced in the resonance fields of non-irradiated and irradiated samples.

Irradiation-generated changes in the magnetic behavior and in the mechanical properties were measured and compared in RPV forging and weld surveillance Charpy specimens to reveal the possible correlations between them [78]. The samples were irradiated by E > 1.0 MeV energy neutrons up to the fluence of 2.3 × 10^19^ n/cm^2^. Tensile and Charpy impact tests and Vickers microhardness measurements were carried out as mechanical parameters. Magnetic parameters, such as saturation magnetization, remanence, coercivity, and Barkhausen noise amplitude were determined for non-irradiated and irradiated specimens. Hysteresis loops were found to turn clockwise, which resulted in an increase in coercivity. The Barkhausen noise amplitude decreased after irradiation. These magnetic parameters revealed a correlation with the changes in mechanical parameters.

Recently, research on magnetic methods to investigate the neutron-irradiation embrittlement processes of RPV steel has been focused mainly on Barkhausen emission measurements and on minor hysteresis loop measurements, as will be discussed in the next subsections. 

### 4.2. Barkhausen Emission Measurements

Promising results were also achieved in the field of magnetic nondestructive tests of irradiated RPV steel by applying Barkhausen emission measurements. One of them was already mentioned in the previous sub-section [59].

As shown in [79], the measurement of Barkhausen emissions can reveal neutron-irradiation-caused degradation in a pressure vessel; this parameter was in the range of −20% to −45% in the case of fluences up to 25 × 10^18^ n/cm^2^.

MBN and magnetomechanical acoustic emission were applied in Mn–Mo–Ni pressure-vessel steels having different microstructures for studying the influence of microstructural changes on these parameters [80]. The measured signals were significantly affected by the microstructural features. The MBN energy varied inversely with hardness and it also depended on the microstructure. The results indicated that these quantities were closely related to the dislocation density and residual stress.

Barkhausen noise measurements were used for the investigation of the radiation damage and thermal recovery of irradiated RPV steel samples [81]. There were two recovery stages identified from the hardness measurements. This effect resulted from isochronal annealing, and it was presumed to be accountable for it. The mechanism can be explained by using the results of MBN measurement on the basis of the interaction between radiation-induced defects and the magnetic domain wall. Irradiation caused an increase in the maximum magnetic induction, but the coercivity was not modified by neutron irradiation. The MBN parameters associated with the magnetic domain wall motion decreased due to neutron irradiation, and they recovered with subsequent heat treatments.

The papers [73,82] deal with the use of MBN measurements to study the irradiation effects on nuclear reactor structural materials. Different RPV materials were investigated, and the specimens were irradiated with different neutron fluences. A stabilized flux mode was used, and the magnetic flux within the sample was controlled to compensate for leakage and variations with the flux. An anisotropy effect caused by the sample cutting direction was found, which masked the magnetic signal induced by the irradiation effects. Specimens cut in the same direction correlated with irradiation-generated material hardening and its dependency on fluence. It was found that different materials resulted in different hardening levels. MBN parameters were correlated with neutron fluence by taking into account the cutting direction.

The microstructure effects on MBN emission and on first-order reversal curve (FORC) analysis were studied in a ferritic/martensitic alloy (HT-9), which can be interesting for experts in nuclear materials [83]. It was found that MBN emission and the reversible component of magnetization, determined from the FORC data, decreased with increasing mechanical hardness. The results were discussed in terms of the use of magnetic signatures, for use in NDE, of radiation damage and other microstructural changes in ferritic/martensitic alloys. It was also shown in [83] that FORC analysis is particularly useful for the characterization of defect density and pinning, which can be correlated with bulk NDE field measurements such as MBN emission.

### 4.3. Measurement of Minor Hysteresis Loops

By applying the magnetic minor loops power scaling laws (PSL), the neutron-irradiation-generated modification of minor loops was found in pure Fe and in different model alloys [84]. The minor hysteresis loop coefficients which were determined from scaling relations between minor-loop parameters and in proportion to internal stress decreased in all materials, irradiated by a neutron fluence of 3.32 × 10^19^ n cm^−2^. The decrease in the coefficients was found to be larger for alloys containing Cu, and it was enhanced by a 1% Mn addition. This decrease could have been caused by the reduction in internal stress during irradiation. It is in contrast with the changes in yield strength after neutron irradiation, which increased with Cu and Mn contents. In this paper, a qualitative explanation can be found on the basis of the preferential formation of Cu precipitates along pre-existing dislocations, which decreases the internal stress of the dislocations.

The same PSL method was used for the measurement of low-carbon steel and Fe metal, which were irradiated at 563 K in a 50 MW nuclear reactor [85]. Special attention was devoted to minor-loop coefficients investigating the nucleation mechanism of copper precipitates and dislocation loops during neutron radiation. The minor-loop parameters were found to be very sensitive to lattice defects, such as dislocations, copper precipitates, and grain boundaries. The minor-loop coefficients increased monotonously with an increase in neutron fluence in Fe metal. Here, the dislocation loops have an important role in the brittleness.

Minor magnetic hysteresis loops were also measured on A533B-type RPV steels having various combinations of Cu and Ni content. Samples were irradiated by neutrons to a fluence of up to 3.32 × 10^19^ n cm^−2^ [86]. There was a strong compositional dependence found in the minor-loop coefficient, which is obtained from a scaling power law between minor-loop parameters. This is due to the internal stress. A large increase was found in the low-fluence regime (below 0.4 × 10^19^ n cm^−2^) in the properties of high-Ni and high-Cu steel, which was followed by a slow decrease. However, in low-Ni and low-Cu content steel, a sudden decrease was experienced. These variations are mostly in a linear relationship with changes in yield strength. The results were interpreted from the viewpoint of the formation and growth of Cu-rich precipitates and/or fine scale defects in the matrix and along pre-existing dislocations. A model analysis, assuming the Avrami-type growth of Cu-rich precipitates and an empirical logarithmic law for relaxation of residual stress, demonstrated that the increment in the coefficient due to Cu-rich precipitates increased with Cu and Ni content and was in proportion to a yield stress change, related to irradiation hardening [87,88].

In [89], the applicability of MAT, an alternative method of minor hysteresis loop measurement, is demonstrated for the inspection of neutron-irradiation embrittlement in RPV steels. Three series of samples (JRQ, 15CH2MFA, and 10ChMFT type steels), irradiated by E > 1 MeV energy neutrons with a total neutron fluence of 1.58 − 11.9 × 10^19^ n cm^−2^, were investigated by this method. Correlation was demonstrated between the MAT parameters and the neutron fluence in all types of the investigated materials. The shift of the DBTT as a function of the neutron fluence for the 15CH2MFA type material was also evaluated. In this case, a sensitive linear correlation was found between the DBTT and MAT parameters. Based on these results, MAT was shown as a promising complimentary tool of the destructive tests within the surveillance programs, currently used for the inspection of the neutron-irradiation-generated embrittlement of RPV steels. It was found that the sensitivity also depended on the structural anisotropy due to the original direction of the material rolling/forging. The samples magnetized along the original direction of steel rolling provided the most sensitive results. Another conclusion of these measurements was that the magnetic properties of the samples were affected by the quality of the magnetic contact between the sample surface and the attached soft magnetic yoke. It was concluded that if the yoke was not in good contact with the sample surface, the results would be strongly influenced.

In other work [90], three methods (MBN emission measurements, PSL, and MAT) were compared with each other on the same series of neutron-irradiated RPV steel material. The JRQ and 15Kh2MFA material and 10KhMFT type welding steels (for WWER 440-type Russian reactors) were used for the measurements, which were irradiated by high-energy (>1 MeV) neutrons with up to 11.9 × 10^19^ n cm^−2^ fluences. MAT was found to be the most sensitive method among the investigated techniques. It revealed the most straightforward linear correlation with the independently measured DBTT values. The other magnetic methods (MBE and PSL) were found to be less sensitive than MAT, and also, they did not correlate with the DBTT, nor with each other. Considering that both MBN and PSL are highly structure-sensitive magnetic methods, the lower sensitivity and their poor correlation with the DBTT can be primarily attributed to the different sensitivity to lattice defects from that of the DBTT. It was also concluded, similarly to the experiences in [89], that an uncontrolled fluctuation of surface quality of the slightly corroded specimens made the measurement difficult. Contactless methods of investigating more conveniently shaped irradiated nuclear pressure-vessel steel samples were suggested for future inspections.

The influence of the rough surface on MAT measurements was carefully analyzed in [91], and it was found that this harmful effect could be significantly reduced by applying a non-magnetic spacer between the sample surface and the magnetizing yoke. 

### 4.4. Electromagnetic NDT Techniques

The 3MA method (micromagnetic, multiparameter, microstructure and stress analysis) has been found to represent a suitable tool for the characterization of the degradation of ferromagnetic materials (like RPV steels).

The microstructure of the steels is modified by the neutron-induced embrittlement. This phenomenon depends on the neutron fluence but also on the special design of the RPV of nuclear power plants. Embrittlement contributes to the increase in hardness and strength on the basis of vacancies and Cu-rich precipitates and also to the shift of the DBTT to higher temperatures. Micromagnetic investigations were conducted on full Charpy specimens and material of the last generation of German NPPs in order to characterize the material degradation [63]. This contribution reports on the results obtained by the application of the 3MA method and the magnetostrictive excitation of ultrasound using an EMAT. Both technologies document potential to be further developed as an in-service inspection technique. 

By using the electromagnetic nondestructive techniques, the ability to characterize material ageing was demonstrated [92]. Thermal degradation causes hardness enhancement and the appearance of Cu precipitation. An early warning can be conducted before fatigue life is elapsed due to low cycle fatigue. 

The DBTT shift indicated when material degradation was present in well-defined laboratory-type specimens. A high sensitivity and confidence in the results was obtained. As the special application of EMAT sensors demonstrated their reliable use at a service temperature of 300 °C, the integration of this sensor type into plant lifetime management systems presents an engineering problem. The purpose is to solve the proper selection of cooling devices for the driving microelectronic systems and to apply heat-resistant wires for coils and cables especially isolated for high-temperature access.

## 5. NOMAD Project

As outlined in the introduction, the long-term operation (LTO) of existing nuclear power plants (NPPs) has already been accepted in many countries as a strategic objective to ensure an adequate supply of electricity over the coming decades. In order to estimate the remaining useful lifetime of NPP components, LTO requires reliable and accurate tools. To achieve a step forward in this important area, a joint research project was initiated by ten different laboratories from seven different European countries. The project (Nondestructive Evaluation System for the Inspection of Operation-Induced Material Degradation in Nuclear Power Plants, or, NOMAD for short) has received funding from the Euratom research and training program 2014–2018 under grant agreement No. 755330. The common work started in 2017 [93].

The primary goal of NOMAD was to develop, demonstrate, and validate an NDE tool suitable for the local and volumetric characterization of the embrittlement in operational reactor pressure vessels (RPVs). The project comprised the following main tasks:

- Development and demonstration of an NDE tool suitable for the characterization of RPV embrittlement, with special respect to the material heterogeneities, and which can exceed the existing information from the current surveillance programs;
- To extend the already-available database of RPV material degradation by adding correlations of mechanical, microstructural, and NDE parameters, as well as including quantifications of reliability and uncertainty;
- Application of the developed tool directly to cladded material, considering the aspects of the on-the-spot applications.

The priorities of reactor operation were taken into account in the realization of the NOMAD project. The approach developed in NOMAD delivers information that can be used complementary to and exceeding the information obtained by standardized destructive tests of surveillance samples. These surveillance samples are currently assumed to represent the whole component. However, these do not take into account the possible local material variations that originate from the production issues or from other local impacts during the operation or maintenance periods. Therefore, NOMAD’s aim was to establish a better, more reliable base level for nuclear safety in the framework of assessment for lifetime operation.

In the previous section, different application possibilities of several novel—mainly magnetic—nondestructive methods were reviewed. As can be seen, good results were achieved in how these methods could be applied for the inspection of the neutron-irradiation-generated embrittlement of RPV steel. However, only a few of them were conducted on mechanically intact RPV steel, which would form a continuous series from non-irradiated samples up to samples of the same RPV material having the same shape, which would be subjected to a high fluence of neutrons in a nuclear reactor. Hence, it can be stated that presently there is no real effective nondestructive method for radiation embrittlement evaluation in practical use. One of the reasons for the contradictory results is that the investigated irradiated specimens were mostly collected from different surveillance programs. Furthermore, the investigated specimens were often mechanically deformed, sometimes corroded, and in several cases the zero-level specimens were missing or they were cut from different parts of the material.

Within the NOMAD project, several different nondestructive techniques were applied to detect the neutron-irradiation-generated material degradation in many different RPV steel materials, having either Charpy geometry or larger cladded or non-cladded blocks. The same series of samples were measured before and after neutron irradiation, and the results obtained by different methods were compared with each other. The purpose was to reveal the benefits and drawbacks of different methods, and finally to find which techniques are suitable for future practical applications. The parameters extracted from different methods were compared with the destructively measured DBTT values. A combination of several NDE methods has been used to examine how accurately and reliably the degradation of RPV material can be determined, especially when compared to sample-based destructive testing.

Charpy samples and cladded and non-cladded blocks of various RPV steels and irradiation states were provided. NDE techniques were adapted and applied to measure the irradiated and non-irradiated samples. The applied NDE methods were the magnetic methods MAT, 3MA-X8, and MBN, the electrical methods DCRPD and TEP, and the ultrasonic method Piezo-US. They were used to characterize the microstructure changes and the variation in the material properties as a function of progressing exposure (fluence level). Table 1 gives an overview on the NDE methods applied to different specimen geometries (Charpy and blocks) within the project.

Destructive reference tests for the determination of the DBTT and other material characteristics were carried out. The irradiated samples were measured and the data were correlated with the DBTT values by using machine-learning methods.

The ultimate goal was to produce a machine-learning-based computational tool that can estimate the neutron-irradiation-induced embrittlement of reactor pressure-vessel steel alloys based on the NDE parameters. This would revolutionize the current surveillance procedures, which rely heavily on destructive methods.

The nondestructive characterization was carried out by means of micromagnetic, electrical, and ultrasonic methods at various stages of material degradation caused by neutron irradiation. They were used to characterize the microstructure changes and the variation in the material properties as a function of progressing exposure (fluence level). Table 1 gives an overview on the NDE methods applied to different specimen geometries (Charpy and blocks) within the project. 

In the following chapters, the results of the NOMAD project will be presented and analyzed.

## 6. Materials

Several sets of samples with various irradiation and embrittlement conditions were provided. The first specimen set consists of ISO-V Charpy samples of the four materials 18MND5 (weld), 22NiMoCr37, HSST-03 (A533-B), and A508-B. These were existing samples from previous CHIVAS surveillance programs, which were available in non-irradiated states and in two or three irradiation states. The second specimen set also consists of ISO-V Charpy samples of the two materials A508Cl.2 and 15kH2NMFA. The A508Cl.2 was cut out from the ¾ depth of a large part of the Lemoniz reactor vessel, a Spanish reactor of Western type that was never operated [94,95]. The 15kH2NMFA material was cut out from an original eastern 1000 MW RPV from the ¼ depth. The 15Kh2NMFA (CrNiMoV) forging steel was manufactured by the Russian IZHORA company for a 1000 MW WWER reactor. The original heat number is “181358” and the forging steel was produced according to the Russian specification TU 108.765-78. These Charpy samples were irradiated at three well-defined levels at the Belgium Reactor BR2. The neutron irradiation was performed in a specially designed rig where 24 Charpy specimens were directly irradiated [96]. The second set of specimens could be nondestructively measured before and after irradiation, so an evaluation of the progressive change in the embrittlement could be performed. Table 2 shows the corresponding material compositions.

**Figure 2:**
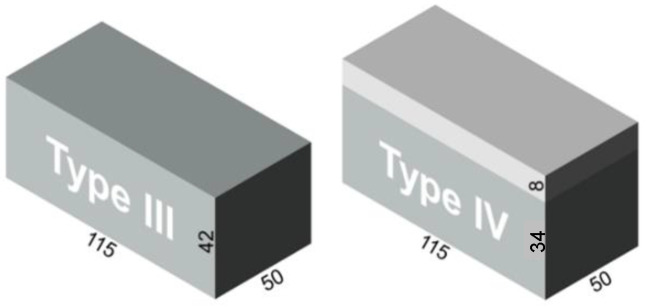
Dimensions (in mm) of the non-cladded and cladded block samples, respectively.

Finally, in order to characterize the neutron-irradiation-induced embrittlement through the cladding, a third specimen set was chosen consisting of six cladded and six non-cladded blocks of Western RPV material A508 Cl.2 (see also Table 2). They are more representative for in situ vessel inspection than the standard test Charpy samples and have been fabricated and irradiated over a large range of neutron fluences at BR2. One part of the block samples without cladding was made of compact material cut out of the mid area of a real RPV, whereas another part of the block samples with cladding was cut out from the cladded surface of the RPV. Thus, the latter can be used to examine the capability of the nondestructive measuring methods for testing the irradiation-induced material degradation through the cladding. The dimensions of both types of block samples are shown in Figure 2.

These blocks were irradiated at three defined levels within BR2 similarly to the second specimen set. Two different strategies have been applied for the irradiation. The first one aimed to achieve an as-uniform-as-possible neutron fluence profile and was applied on three cladded blocks and three non-cladded blocks. The second one aimed to achieve damage that is much more realistic since the blocks have experienced a fluence attenuation profile.

## 7. Mechanical Testing

Charpy impact tests were performed on all of the samples previously investigated nondestructively in order to determine the DBTT. For the determination of the DBTT of the blocks, Charpy samples have been cut out from the top layer, as well as from the bottom layer. By this procedure, individual DBTT values for both sides of the block specimens could be provided. The results of the mechanical testing for all three specimens sets are shown in Table 3, Table 4 and Table 5. 

For the CHIVAS samples (first sample set), it is visible that the DBTT is not strongly correlated with the real fluence level. Probably, the overlapping varying fluence temperature may cause an annealing effect, which reduces the influence of the irradiation. 

For the Charpy and block samples (second and third sample sets), where the irradiation temperature was constant it can be seen that there is a strong correlation between the fluence level and the DBTT for the samples with zero, low, and medium fluence. However, for the samples with medium and high fluence there is no further increase in the DBTT. There is a clear saturation effect.

## 8. Experimental Results

### 8.1. Measuring Procedure

In order to guarantee reproducible measurements and a stable sample/probe coupling, sample/probe holders were constructed for each NDE technique. These were relevant especially for measuring the irradiated samples in the hot cell by the mechanical manipulation for handling and positioning the samples onto the NDE probe.

In order to define a unique way to perform all NDE measurements, an inspection and quality assurance procedure was defined. According to this procedure, each sample was measured five times, each time picking up the probe and replacing it on the sample. Although sample/probe holders were used, the remaining scattering caused by the handling could be averaged by the repetitions. 

As the measuring activities were spread over three years, it was necessary to monitor the stability and functionality of the measuring NDE devices and probes. Therefore, calibration samples were defined and were periodically measured by NDE. The analysis of the resulting outcome of these NDE measurements yields to the conclusion that all NDE results were stable over time. 

### 8.2. Description of the NDE of Embrittlement

#### 8.2.1. Charpy Specimens

**Figure 3:**
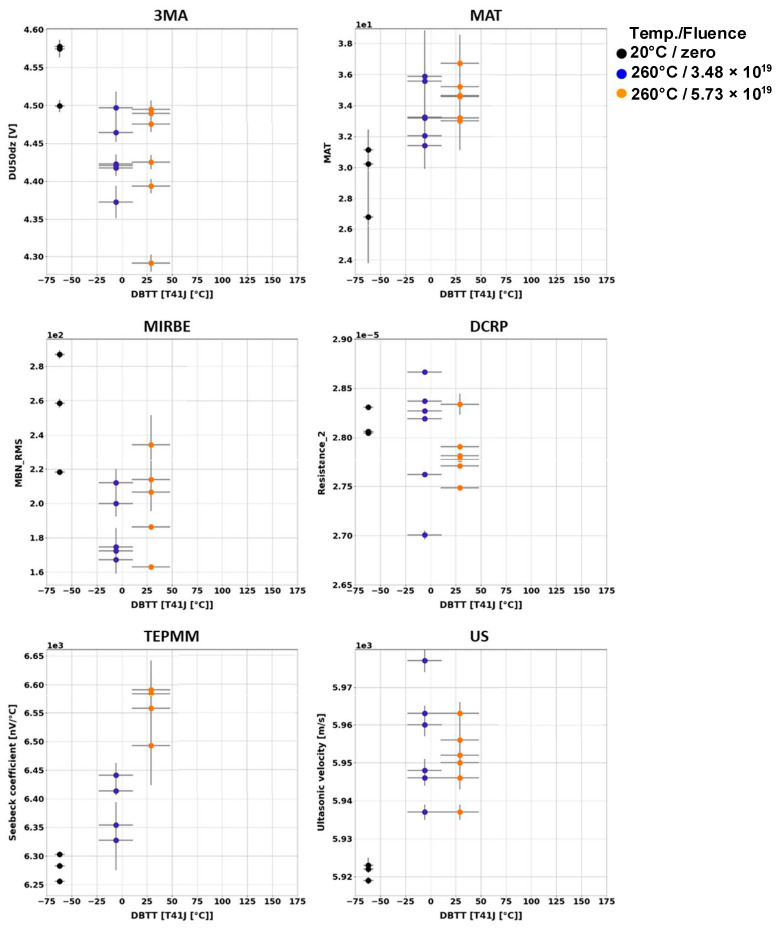
NDE features measured on Charpy samples of 22NiMoCr37.

**Figure 4:**
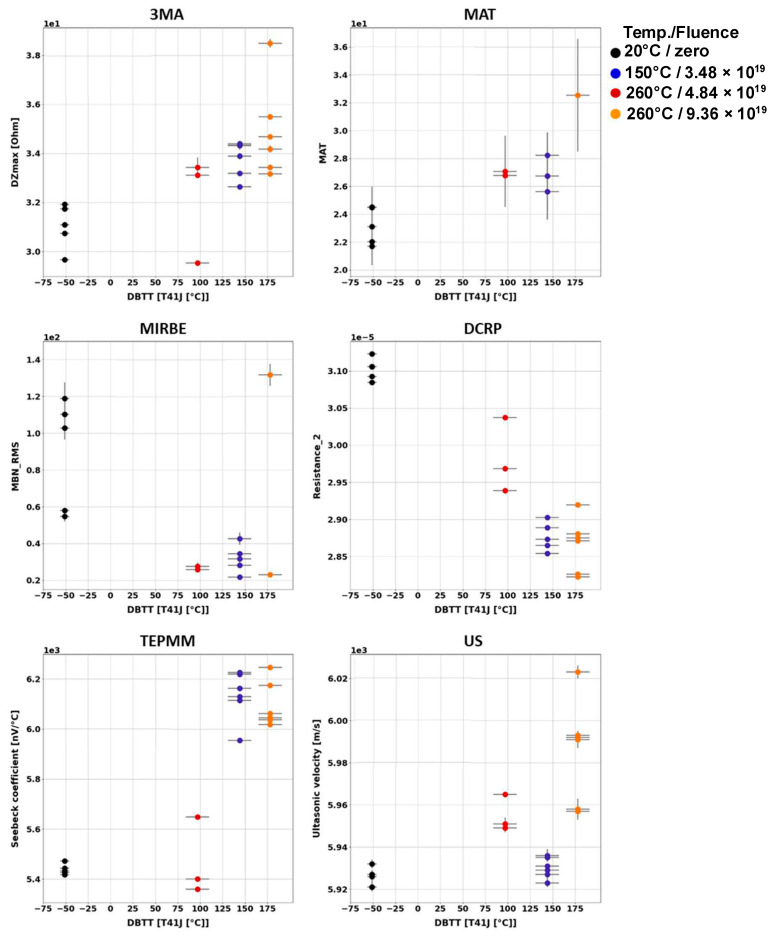
NDE features measured on Charpy samples of 18MND5-W.

**Figure 5:**
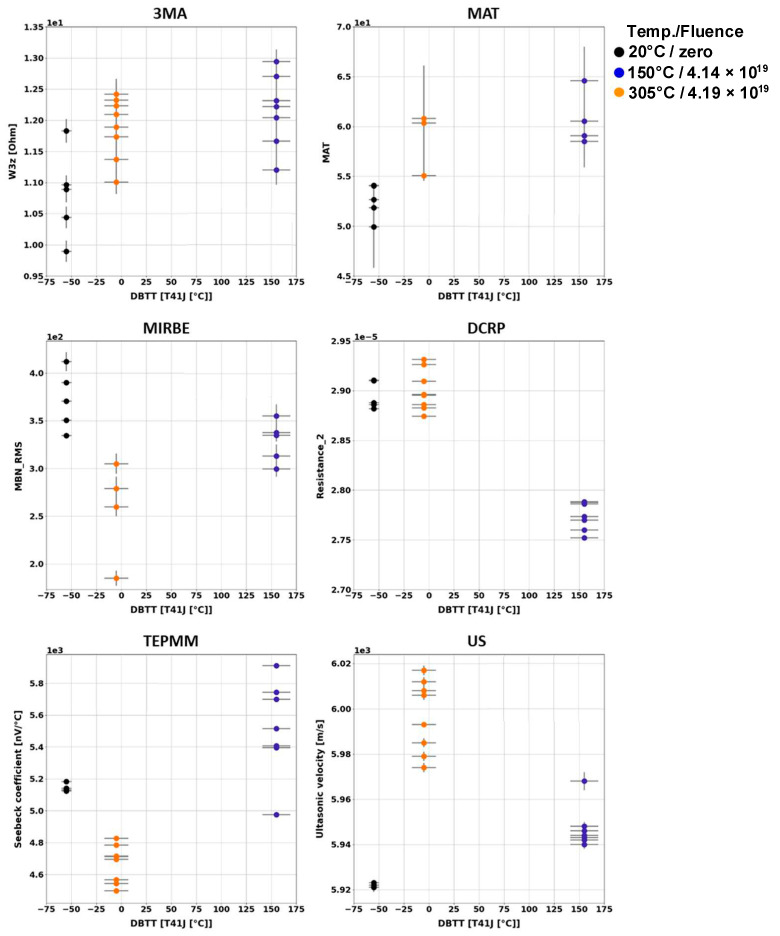
NDE features measured on Charpy samples of A508-B.

**Figure 6:**
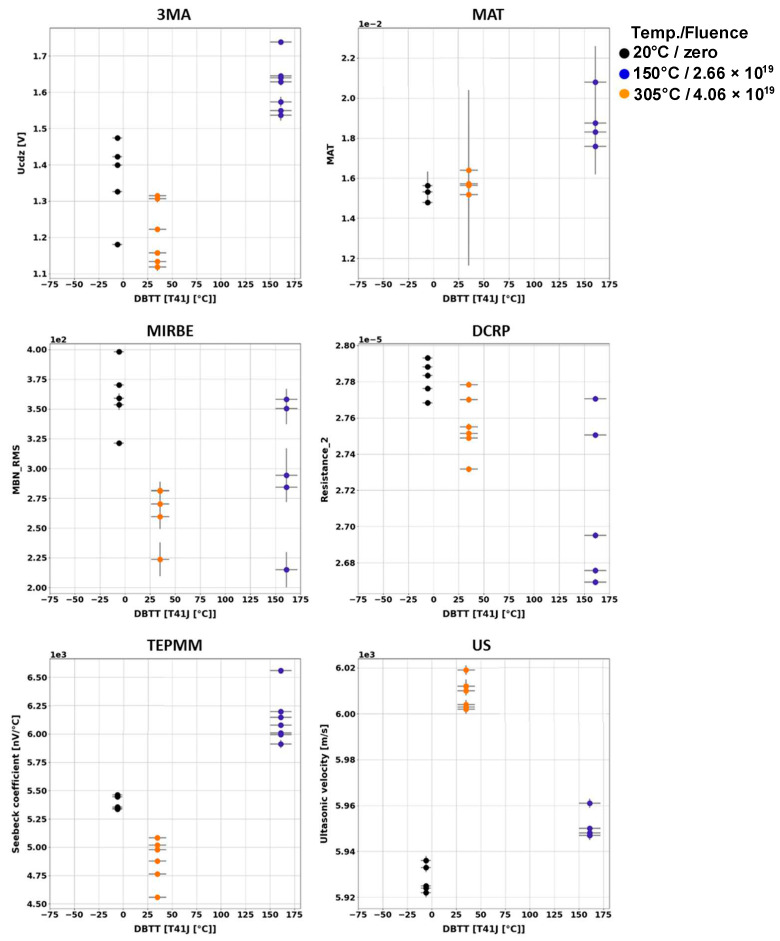
NDE features measured on Charpy samples of HSST-03.

**Figure 7:**
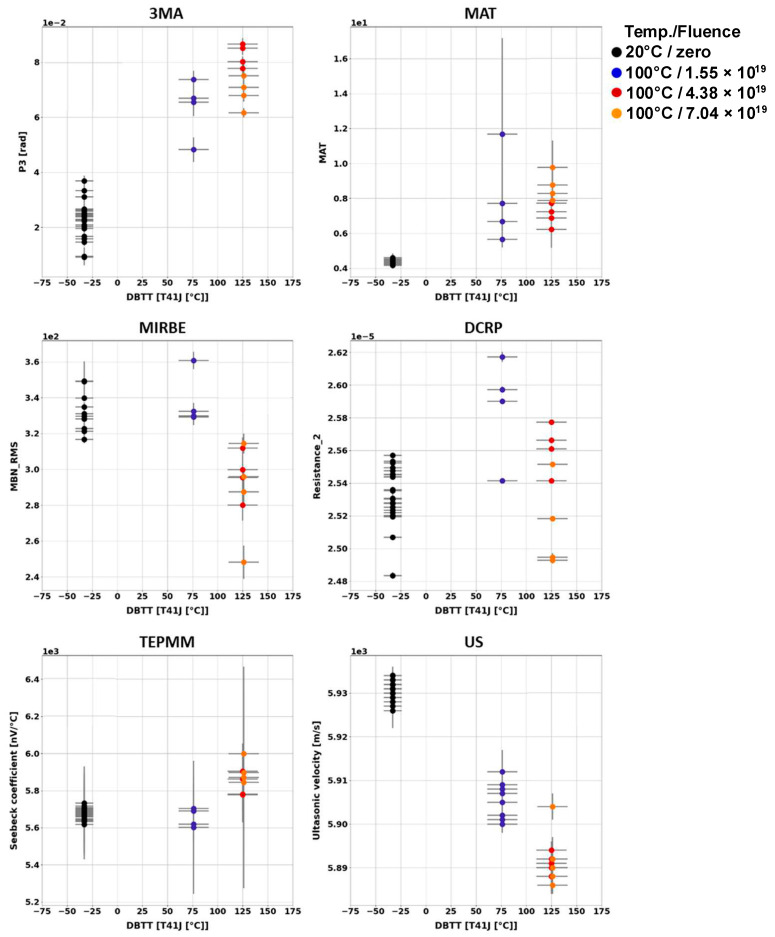
NDE features measured on Charpy samples of A508 Cl.2.

**Figure 8:**
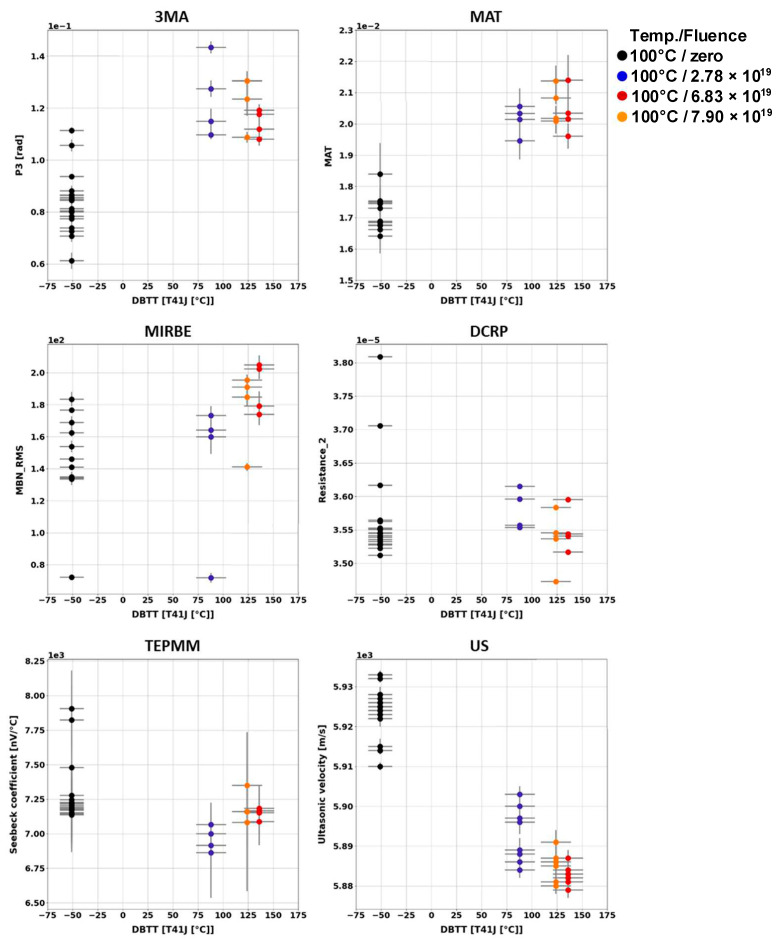
NDE features measured on Charpy samples of 15kH2NMFA.

Generally, it has been observed that the outcome of the individual NDE measurements performed on different Charpy samples of the same material and the same irradiation condition scatter. Figure 3, Figure 4, Figure 5, Figure 6, Figure 7 and Figure 8 give an overview about the measuring results for each set of Charpy specimens and for each NDE method depending on the corresponding DBTT. A possible explanation of this scattering lies in the different origin of the samples having the same irradiation condition and, consequently, slightly different material properties before irradiation. (This effect is discussed in detail in Appendix B). This causes different progress of the material properties during neutron irradiation. Long-term repetition measurements carried out during the measurement campaigns on a reference sample showed stable results. Uncertainty studies were performed in order to find out the origin of this scatter. These studies concluded that the uncertainty of the samples can be estimated to be higher than the uncertainty of the “inspection system” [97]. Nevertheless, the correlation of the nondestructively extracted features with embrittlement has been identified. Several trends correlate with each other and deliver complementary information regarding the material properties. It was expected that the NDE features showed either a continuously increasing or a continuously decreasing trend for a certain material. This behavior cannot be confirmed in the case of all materials (see Figure 6—material HSST03).

Moreover, it was observed that the same NDE feature shows different trends for different materials: the Seebeck coefficient is expected to increase with embrittlement. However, it decreases in the case of the materials A508b and HSST03 irradiated at 305 °C compared with the non-irradiated condition. 

The behavior of the NDE features is affected by both the irradiation fluence and the irradiation temperature. The increase in the fluence causes an increase in the embrittlement. The increase in the temperature has different effects on some of the NDE features: whereas temperatures below 260 °C have the same influence like the irradiation fluence, it seems that higher temperatures have a contrary effect especially for the ultrasonic (US) method and Seebeck coefficient measurement (TEPMM). 

In the case of the 3MA method, only the most significant feature of the 21 measured features are shown. (In these figures, the terminology “Micromagnetic Inductive Response and Barkhausen emission (MIRBE)” is used for the Barkhausen noise measurements).

In the case of the material 22NiMoCr37 (irradiated between fluences of 3 × 10^19^ n cm^−2^ and 6 × 10^19^ n cm^−2^), only the Seebeck coefficient continuously changed with an increasing DBTT. No difference in terms of the other NDE parameters was observed for both irradiated conditions (Figure 3). The variation in the DBTT due to increasing fluence, taking into account the scatter of the points of the 95% confidence bounds of the Charpy test results, could not be detected accurately, probably due to variations in the microstructure of the material before irradiation.

In the case of the weld material 18MND5-W (Figure 4), several NDE features show continuous trends. However, in the case of this material, the ultrasonic time-of-flight (TOF) of the samples irradiated at 150 °C is comparable with the TOF of the non-irradiated samples. 

In the case of the materials A508-B (Figure 5) and HSST03 (Figure 6), the high irradiation temperature of 305 °C affects the outcome of the NDE measurements. Especially the results of the methods MIRBE, Piezo-US, and TEPM show an anomalous behavior compared with the other irradiation conditions.

The NDE measurements on the Charpy sample sets of RPV materials of A508Cl.2 (Figure 7) and 15kH2NMFA (Figure 8) investigated before and after irradiation allowed for the first time the characterization of progressive change in embrittlement taking into account the initial condition of the materials (before irradiation). In the case of this set of samples, it has been observed that the scattering of the outcome of the individual NDE measurements performed on different Charpy samples of the same material and the same irradiation condition is lower. 

The influences of neutron irradiation on several NDE parameters of Charpy specimens have been previously published and discussed in detail [98,99].

#### 8.2.2. Cladded and Non-Cladded Blocks

One of the challenging tasks of the NOMAD project was to develop a method that can follow the degradation of the base material through the cladding, i.e., the probe can be applied from the inside of the reactor vessel wall. This task raises two problems simultaneously: On one hand, to find a way to excite the base material through the cladding, and also to detect the response through the same. On the other hand, the degradation of the base material has to be differentiated from the changes in the cladding properties due to the irradiation.

**Figure 9:**
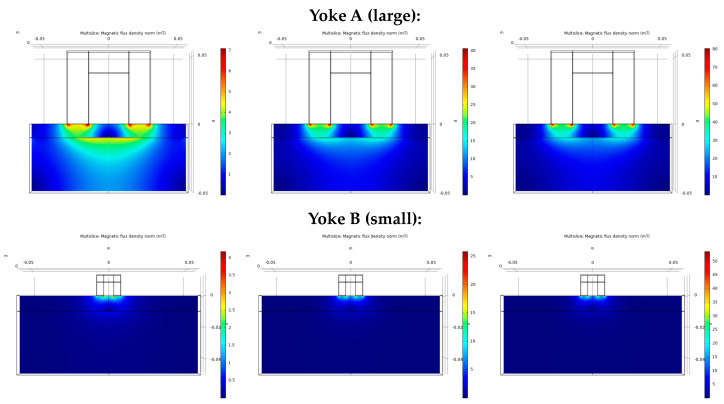
Qualitative collation: calculated distribution of the magnetic flux density in a cladded block excited through the cladding when large and small yokes are placed onto the top of the cladding which has different relative permeability [100].

To decide whether the ferromagnetic base material can be investigated through the austenitic cladding by magnetic methods using an attached magnetizing yoke, numerical simulation of the magnetic field was performed to show how the magnetic field generated by the magnetizing yoke penetrates into the base material through cladding [100]. The result of this simulation is presented in Figure 9. The result of numerical simulation revealed that by using the large magnetizing yoke for generating an exciting magnetic field, the base material could be magnetized sufficiently even through the cladding. On the other hand, when a yoke of a small size compared to the thickness of the cladding is applied, it can mostly excite the region of cladding, and the density of the penetrating exciting field in the base material is very limited, almost negligible. In this way, the cladded system and the cladding only can be studied separately.

To characterize the neutron-irradiation-induced embrittlement independent of the initial microstructure that can be heterogeneous, all blocks have been nondestructively investigated by means of all previously optimized NDE methods before and after neutron irradiation. Some NDE methods (DCRP and EMAT) are not suitable for the materials’ characterization through the austenitic cladding. 

NDE measurements carried out before neutron irradiation have shown that, similar to the Charpy samples made of the same material, the blocks (non-cladded or cladded) have different material properties. The explanation therefore lies in the different microstructure at different positions in the component where these investigated blocks originate from. 

**Figure 10:**
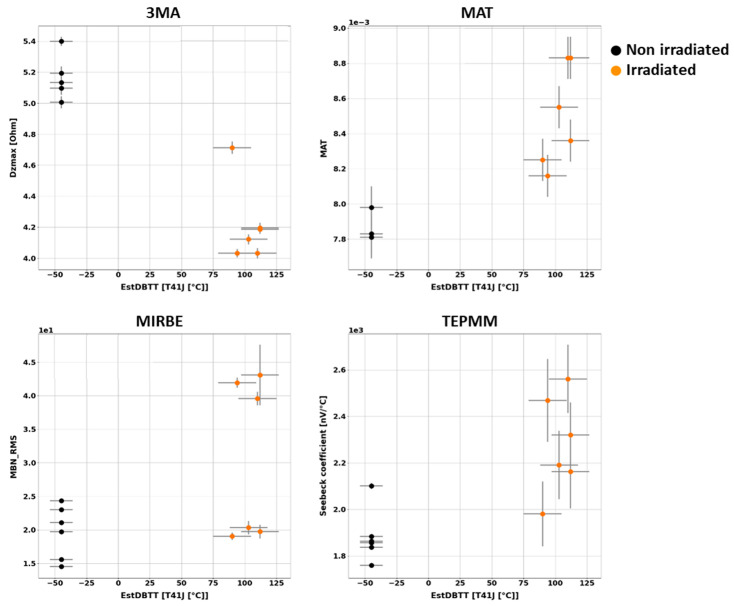
NDE features measured on cladded blocks through the cladding.

**Figure 11:**
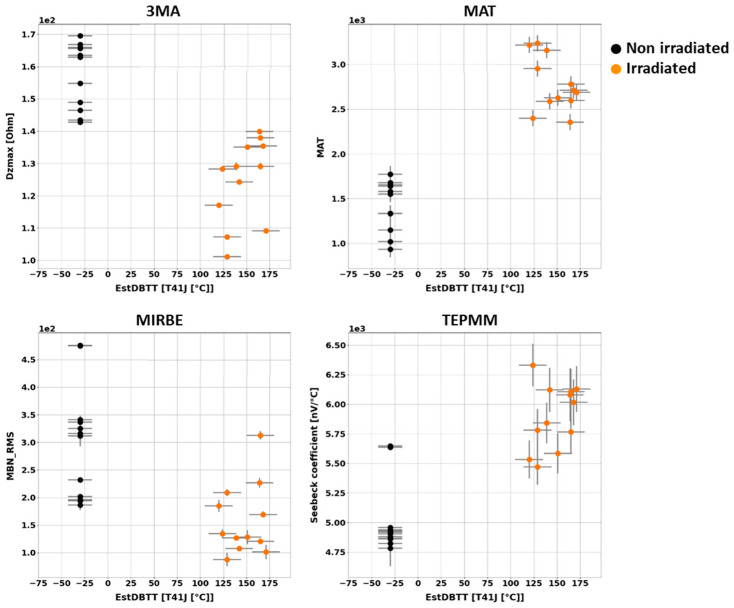
NDE features measured on non-cladded blocks A508 Cl.2 on two opposite sides.

After irradiation, NDE measurements have been carried out again on all non-cladded and cladded blocks. Measuring quantities derived from the NDE methods have been individually collected and analyzed in terms of the DBTT. The trends of the nondestructively determined measuring quantities have been identified. An overview of the results obtained on the block samples is given below (Figure 10 and Figure 11). 

The influences of neutron irradiation on the MAT parameters of block specimens have been previously published and discussed in detail [101,102].

The results of the individual NDE methods have shown difficulty in characterizing the embrittlement using single-parameter methods. 

## 9. Results for Prediction of Embrittlement by Means of a Multi-Parameter Machine-Learning (ML)-Driven Approach

### 9.1. Multiparameter Method

The individual test quantities have differently weighted sensitivities to target and disturbance quantities. This creates an opportunity to recognize and to suppress the influence of the disturbance quantities by combining these quantities. The advantages of combining test quantities in a multi-parameter method are manifold. Such a combination of methods is particularly indispensable when the target quantities, so-called independent parameters, are to be predicted (e.g., DBTT) and the disturbance quantities (temperature, residual stresses, surface condition) can vary simultaneously. The ultimate goal was to produce a machine-learning-based computational tool that can estimate the neutron-irradiation-induced embrittlement of reactor pressure-vessel steel alloys based on the NDE parameters.

In the case of multi-parameter, i.e., multi-dimensional nonlinear problems, the required analytical expression cannot be formulated typically, so another statistical approach is required. ML algorithms are powerful tools that can be used to automatically create a regression model based on given data. The data must consist of one or several features, which are the input to the model, and one or several target variables, which are the desired prediction of the independent parameters, or in other words, the output of the model.

**Figure 12:**
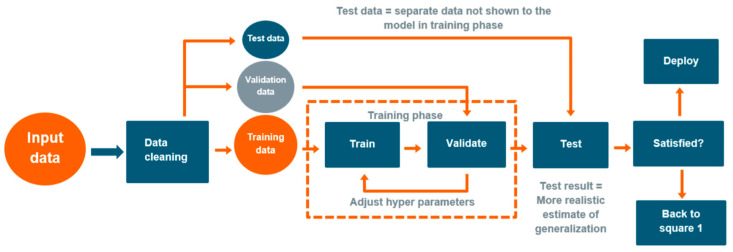
Possible workflow of the ML method application [103].

Since the analytical expression that links the input and the output parameters is not known in the case of ML, transparency is essential for the application of ML, especially in safety areas. Extra attention has to be paid to careful studies of these methods and to their verification. In typical supervised machine learning, the model learns from given training data, and traditionally, a separate validation dataset is used to evaluate the performance of the trained model. But here, an additional dataset is required for testing the reliability of the method. This dataset not only comprises some randomly selected elements but also data selected of the interval boundaries (see Figure 12). When the test result is unsatisfactory, the whole procedure has to be restarted (i.e., “Back to square 1”), by experimenting with new model structure and hyper-parameters.

### 9.2. Database Preparation

Statistical methods work well on a large dataset. This raises a quite frequent problem of ML applications: the insufficient number of the achievable measured samples or experimental data, i.e., the limited size of the true results database. The ML methods do not generate any novel information. Instead, they can be used to circumscribe and recognize the information that can be found in the database. Pre-processing the obtained database of the physical test results aims to ease this task of the ML approach and increase its performance, i.e., increase the precision of the regression or classification. Any operation on the experimental dataset should be carried out with special attention not to degrade the targeted information content or falsify it.

There are basically two options for processing the database: eliminating some of its elements considered as useless ones, and modifying its entries, or even generating novel ones in an artificial way.

#### 9.2.1. Reduction in Input Space Dimension

If the analysis includes too many features that have a weak correlation with the target variable, they can reduce computational efficiency, increase model complexity, and make it harder to interpret. Moreover, increased model complexity results in a higher probability of overfitting. A large number of features can result in a problem known as the curse of dimensionality. By increasing the number of dimensions, the volume of the feature space will be increased as well, which leads to sparser data. As the number of features increases, the number of samples required to maintain accuracy grows exponentially [103].

One of the most evident methods for reducing the dimension of the input space is to remove the least relevant features (i.e., input parameters). This reduces the complexity of the ML algorithm’s problem. There are various simple statistical evaluations or more complex regression methods to study the importance of the different features.

#### 9.2.2. Determining Feature Importance

As an example, the Wilcoxon signed-rank test [104] can be used to study the statistical significance of materials’ properties caused by the irradiation in this paired dataset. This test calculates the synthetic parameter differences for each pair of measurements and separately for each NDE feature. The outcome of the test is the *p*-value for the null hypothesis that the difference has a zero expectation value and a symmetric distribution. This corresponds to the conclusion that the irradiation did not affect the feature. The *p*-value is compared to a predefined significance level: If the *p*-value is greater than the significance level (threshold), the null hypothesis is accepted, and we conclude that irradiation has not caused a change in the feature, which can then be excluded from the analysis.

**Figure 13:**
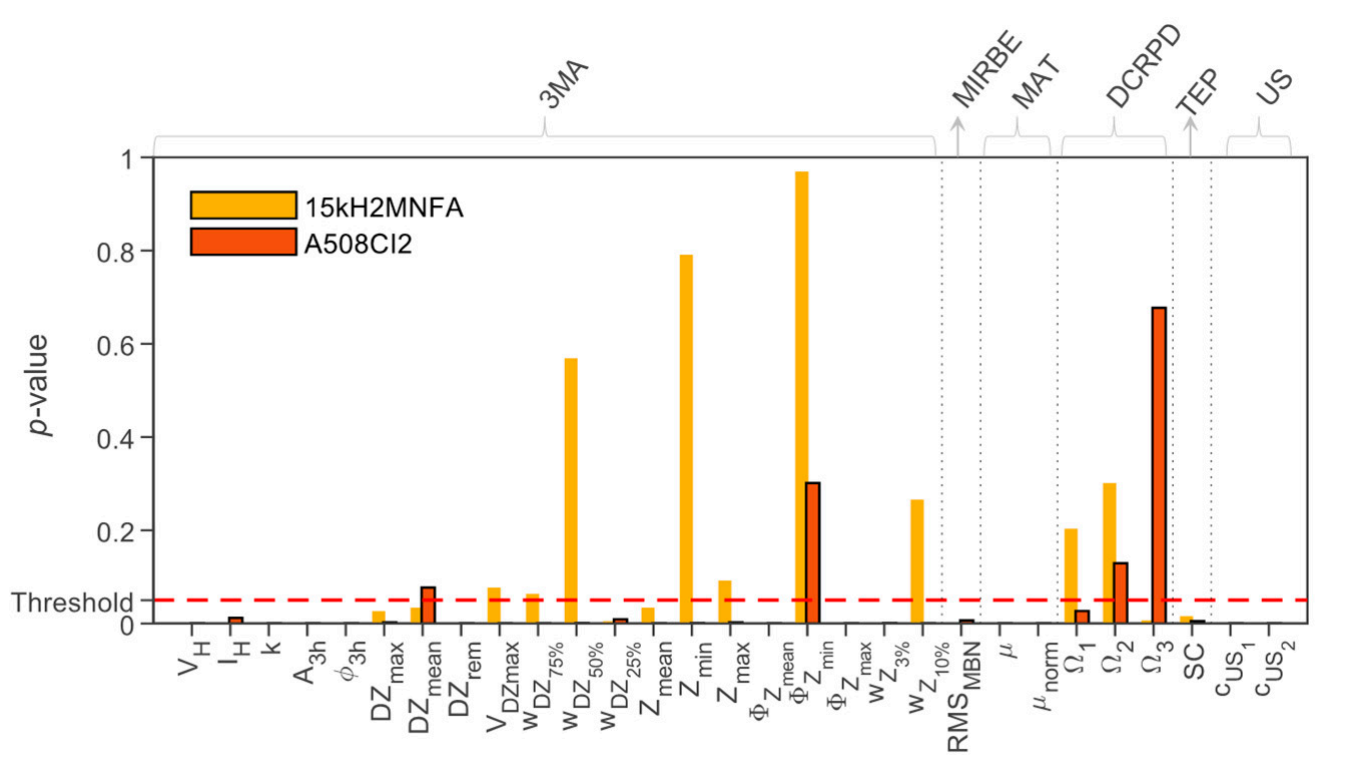
Example: Wilcoxon test results obtained on 29 NDE features (i.e., measurement output parameters) of the NOMAD project [103] (threshold = 0.05).

In the NOMAD project, the analysis focused on identifying which NDE parameters out of the 28 available contribute to precision and can aid in recognizing and suppressing side effects. In addition, the project isolated the parameters that are irrelevant and have no impact. Different types of ML algorithms were tested in a competition-type manner and their performances were evaluated (see the example in Figure 13).

**Figure 14:**
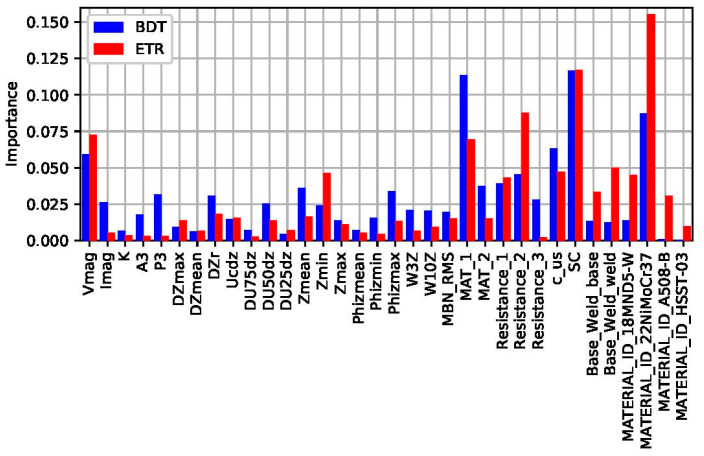
Example: The relative importance of the features evaluated by the models built with the boosted decision tree (BDT) algorithm and the extra-trees regressor (ETR) algorithm plotted as bar plots in the case of the NOMAD database involving six different tested materials [102].

The Wilcoxon signed-rank test is a simple approach that can be applied even on very limited datasets but also with limited accuracy. However, when the dimension of the database allows it, even more sophisticated methods can be implemented to study the importance of the input features. Ensembles of decision trees can be used for this purpose. The boosted decision tree (BDT) algorithm and the extra-trees regressor (ETR) algorithm can be used for better evaluation of the different feature significance (see Figure 14). These methods are typically not for the primary assessment, but rather for optimizing the ML tool to be developed [102].

#### 9.2.3. *k*-Fold Cross-Validation Score

A traditional method to train a machine-learning model is to set aside a third dataset from the training data, known as the testing dataset, that is used to test the different hyper- parameter combinations (i.e., the combinations of those external configuration variables used to manage machine-learning model training), for example. However, the model can also be trained using *k*-fold cross-validation [105], where the training set is divided into *k* parts, known as folds, and then one by one, each fold is used as a so-called test, or validation, set while the other *k* − 1 folds are used to train the model. This is repeated *k* times and an averaged cross-validation score is calculated based on the predictions. Obviously, the separate test set must still exist and should be held out of this training process. There are several benefits to cross-validation: the real test set is not shown to the model at any point of the training process, and the *k*-fold cross-validation score provides an estimate of the test accuracy of the model.

The *k*-fold score is used to fine-tune the data pre-processing steps and the hyper-parameters of the model. That said, it can also be used for determining the less relevant parameters. Dropping one of the features reduces the dimensionality of the feature space and can improve the performance of some algorithms. This way, dropping the one-hot encoded features one by one and calculating the *k*-fold cross-validation score for each feature, the least significant features can be identified and eliminated from the database [106].

### 9.3. Manipulating the Database

In order to increase the achievable classification accuracy of the ML methods, the quality of the otherwise limited database can be improved by manipulating its content. Artificial manipulation of any data obtained from real, i.e., physical experiments sounds surprising at first, since this involves significant risks of falsification and of losing the transparency of the ML method application.

It is quite typical when multiple different measurement principles are utilized for obtaining multidimensional responses on the tested target that some of measurement parameters become invalid in certain measurement cases. Here, invalid means that the related method cannot be applied in the given circumstances or some of the NDE probes fail because of an occurring side effect. As a consequence, the aggregated database will not only be limited due to the limited availability of test cases, but it will also be incomplete: there will be missing elements or existing elements having invalid values in it. Either the missing elements or the invalid elements degrade the classification accuracy.

#### 9.3.1. Cleaning the Database

Database cleaning covers the efforts of identification and marking or eliminating database elements that can be recognized as undoubtedly false or missing before the database is used for training the ML methods. This step is not only an option but rather a necessary step in the practice.

The missing elements, as well as the invalid elements, can be marked in the same way and both types can be considered as holding no information. One of the possibilities in practice is to substitute these values with special number values, which are defined exactly for this purpose: NaN (i.e., not a number) [102]. This way, the database becomes uniform and suitable for further processing in terms of meaning where all of its elements will contain uniformly encoded elements. Inserting or replacing elements to NaNs in the database does not alter the information it holds, but makes it possible to process with standard algorithms and software codes.

#### 9.3.2. Dealing with NaNs

By having NaNs, in the database further steps are available for improving the database for ML. If the elements of a certain test case contain too many NaNs or several of its significant elements are NaNs, such test cases (i.e., database rows) can be eliminated completely from the database since they cannot contribute to training the ML methods. Obviously, if all the measurement cases are deleted, where at least one parameter is missing (so-called list wise deletion), it does not cause any bias in the prediction of the ML methods but dramatically degrades the power of the classification. Therefore, the listwise deletion cannot be applied practically where the database is rather limited.

Instead of deletion, there are also different methods of predicting the missing values by either considering the statistics of its available neighbors, when possible, or filling them automatically by algorithms even with the help of ML techniques that consider global features not local ones.

#### 9.3.3. Data Augmentation and Imputation

The aim of both the data augmentation and the imputation is to increase the classification accuracy [107]. Obviously, extending the database of the measured data with “artificially generated” values involves the possibility of either falsification or establishing a “positive” feedback that can lead to misprediction or misclassification. The database extension has relevance primarily for the training of ML algorithms. This way, the unwanted side effect of the database manipulation can be caught by testing the algorithm that was trained on the extended database and on the database without any extension.

### 9.4. Using ML Methods

In the NOMAD project, following the database pre-processing, the training data were used to train three models: Huber loss regression (HLR) [108], support vector regression (SVR) [109], and an artificial neural network (ANN) [110]. In order to compare the performance of these three methods, the 10-fold cross-validation score is measured as the mean absolute error (MAE). Both test and training scores are reported as MAE, root mean square error (RMSE), and R^2^, i.e., coefficient of determination score. Table 6 summarizes the result of this study:

In certain applications of the NOMAD project, a ±25 °C DBTT shift had to be detected as a defined project requirement. All three of these methods could provide a significantly better prediction of the DBTT shift than this error threshold. It can also be deduced that practically all three methods reached a similar performance, i.e., the test scores varied between 16 and 18 °C. The most complex approach, the ANN, could also perform very well, despite the extraordinarily limited dataset.

Since the ANN requires the largest computational power, this test resulted in the SVR prevailing, as this method provides the optimal balance between the classification accuracy and the required computational power. Certainly, this claim is only valid for the given NOMAD database.

### 9.5. Results of the NOMAD ML Approach

Within the framework of the NOMAD project, an SVR method was used to create models that accurately predict the neutron-irradiation-induced embrittlement, measured as the DBTT, even though the dataset was small. The SVR is a supervised machine-learning algorithm that relies on kernel functions to solve problems that cannot be handled directly by linear classification. The SVR is flexible enough that it provides robust results even though the number of data points are low, and it is considerably simpler to optimize than others, e.g., neural network algorithms [102], to evaluate the performance of the trained models in predicting the neutron-irradiation-induced embrittlement of steel alloys based on nondestructive measurements with two different model evaluation metrics.

#### Test Score in NOMAD

The traditional method to analyze the generalization skill of a machine-learning model is to split the dataset into training and test sets. The model is trained entirely based on the training set, and when the model has been fully trained, the generalization skill of the model is tested using the test set by feeding it to the model without its target values (unforeseen data). After the test set has been shown to the model, the model should not be modified to produce an improved test result. The training process refers to the optimization of the model hyper-parameters, and most of the time it also includes pre-processing steps, such as the selection of the feature-scaling scheme. The trained model makes predictions based on the test set features and the predictions of the independent parameters are compared to the true target values of the test set. The test and training sets must be equally representative of the whole dataset; otherwise, the test score does not represent the generalization fit of the model with the best accuracy. Commonly, the test set is a random subset of the whole dataset, with a size of 20% from the whole dataset. The rest, 80% of the data, then constitutes the training set (this procedure is referred to as the 80/20 split). The larger and more diverse the training dataset is, the more flexible and accurate a result the model will yield.

Three models were generated for the prediction of the embrittlement: one based on data obtained on Charpy specimens only, one based on data obtained on blocks only, and one based on mixed data obtained on Charpy and block specimens. The models to predict the DBTT are based on supervised ML using the SVR model implemented in scikit learn and were trained and tested using the database. The database contains different materials which are marked by one-hot Boolean values in both the database and the tool. The input data are prepared using the Min/Max Scaler function within scikit learn.

**Figure 15:**
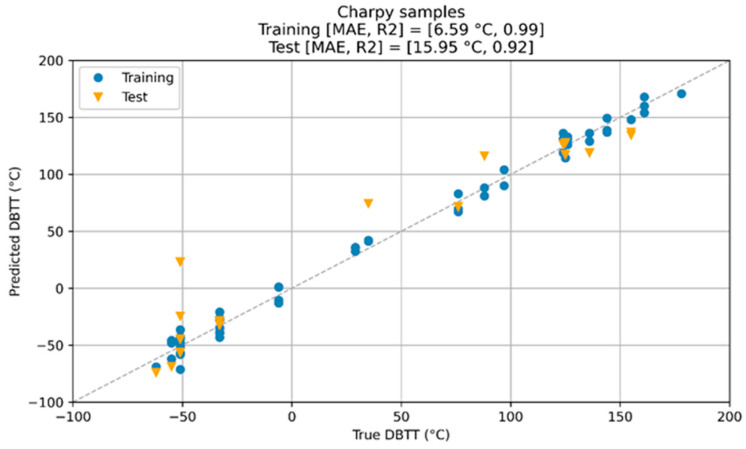
The correlation plot for the Charpy samples. The true values of the ductile-to-brittle transition temperature (DBTT) are plotted on the *x*-axis, and the predictions made by the SVR model are plotted on the *y*-axis. Samples in the training set are plotted as steel blue dots, and samples in the test set are plotted as orange triangles. The grey diagonal line represents a perfect fit. The training and test scores, measured as the mean absolute error (MAE) and R^2^ score, are reported in the heading.

**Figure 16:**
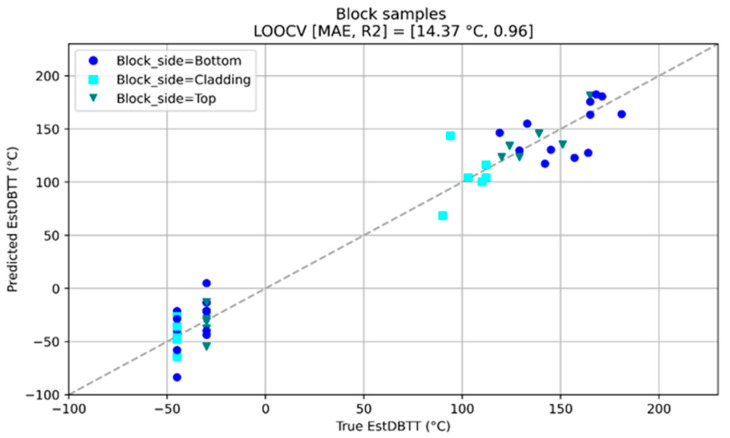
The leave-one-out cross-validation procedure results performed on the entire block dataset, which contains 48 samples. The true values of the estimated ductile-to-brittle transition temperature (EstDBTT) are plotted on the *x*-axis, and the predictions made by the SVR model are plotted on the *y*-axis. The measurements taken from different block sides, top, bottom, and through the cladding have been plotted with different colors and shapes. The LOOCV mean absolute error (MAE) and R^2^ score are reported in the figure heading.

**Figure 17:**
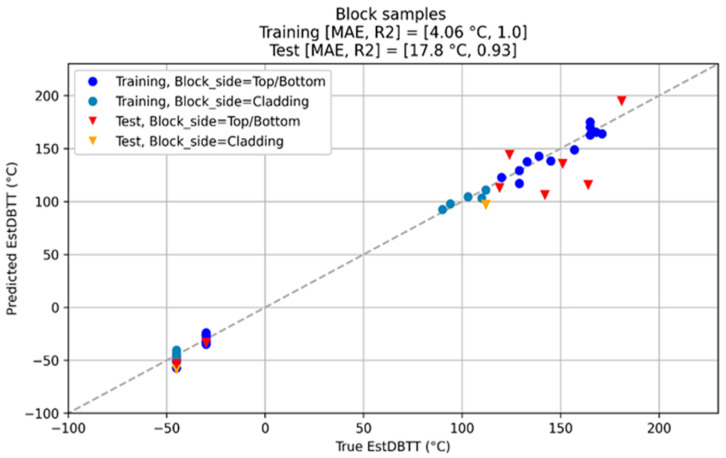
The correlation plot for the block samples. The true values of the estimated ductile-to-brittle transition temperature (EstDBTT) are plotted on the *x*-axis, and the predictions made by the SVR model are plotted on the *y*-axis. Samples in the training set are plotted as blue/steel blue dots, and samples in the test set are plotted as orange/red triangles. Whether the measurement was made through the cladding or not is indicated by the colors. The grey diagonal line represents a perfect fit. The training and test scores, measured as the mean absolute error (MAE) and R^2^ score, are reported in the heading.

The results obtained for the three different models are summarized in Table 7. The table presents the cross-validation scores of the fine-tuned models, the training scores, and the test scores. As can be seen from the results, all test MAEs are under 19 °C and the test R_2_ scores are equal or greater than 0.9. The accuracies of the models are also visualized in the form of correlation plots in Figure 15, Figure 16 and Figure 17, where the predictions made by a model are plotted as a function of the DBTT values determined with Charpy tests.

While the NOMAD study has been conducted on different sample types, including large cladded blocks, the validation focuses on data collected on Charpy samples only due to the limited number of samples available for uncladded and cladded blocks. A transfer of the findings to block types per se is not possible and would require a new validation, respectively. Hence, the validation process is a proof-of-concept regarding the Charpy samples. The validation has been established by a detailed evaluation of the functionality of the NOMAD tool. This has been conducted in the context of the regression as well, as it has been complemented by a classification approach too.

In addition to the training and validation dataset, an additional dataset is introduced for the tool validation. This set remains constant throughout the entire validation process and is not considered for training nor validating. This ensures that the different validation procedures can always be compared through the same dataset. This dataset consists of nine samples and contains only measured NDE values (no data augmentation is used in the validation).

**Figure 18:**
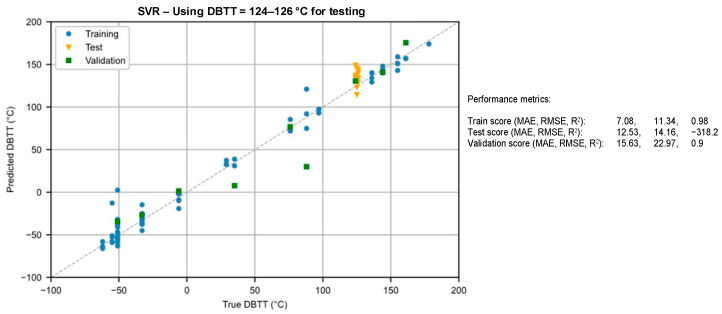
NOMAD tool performance evaluation leaving out the temperature level around 125 °C for training (orange markers).

The robustness of the NOMAD tool was challenged by splitting the test and training set in a way that one temperature level was used for the test set and thus was entirely left out for training. The temperature levels used for testing were 124–126 °C. This analysis gives insight into the question of whether the algorithm is overfitted, as well as its ability to interpolate between different temperature levels. The results are shown in Figure 18. The results show that the NOMAD tool still performs very well, and very good performance metrics are achieved even if one temperature level is not included for training.

## 10. Discussion

A total of nearly 200 non-irradiated and irradiated samples were provided, mechanically tested, and nondestructively measured, and the results were both evaluated and analyzed. 

The sample set consisted of Charpy samples of six different materials and of block-shaped samples of one material, whereby a part of the block samples was covered with cladding in order to simulate the real pressure-vessel situation. In total, 28 NDE parameters out of 6 NDE methods (three magnetic, electric, thermal and ultrasonic methods) were measured from all provided samples. In parallel, the various specimens were tested destructively to determine the irradiation-induced changes in their properties, including tensile, Vickers hardness, and Charpy impact transition curves allowing the determination of the DBTT.

Previously to the NOMAD project, none of the NDE methods or their output parameters could reach the required precision and reliability for application in determining the DBTT. 

It can be noticed, that samples within one material and degradation group show a large variation in the measured feature. This may be caused by influences overlapping with the DBTT caused, for example, by material inhomogeneities, machining variation, or variation in the surface condition. Single features are not suitable for the determination of the DBTT. However, a combination of several features may have the potential to suppress these overlapping disturbance influences. A possible interpretation of the large scatter of the measured points is given in Appendix B. 

The advantages of combining test quantities in a multi-parameter method are presented. This combination of methods has particular relevance when the target quantities to be determined (e.g., DBTT) and the side effects of the disturbance quantities (temperature, residual stresses, and surface condition) can vary simultaneously. Since the individual test quantities are affected by the target and disturbance quantities in a different manner, the influence of the disturbance quantities can be recognized and eliminated or at the very least reduced in this way.

The NDE methods are a kind of inverse problem solution that measure not the targeted physical quantity in a direct manner but other physical quantities that can be linked with it, based on physical background. However, the irradiation effects are complex processes inside the material that affect the recorded quantities in different ways. 

This is why a stand-alone NDE method based on a single physical principle has to face the problem of several side effects resulting in an unacceptable scattering of the output parameter. Therefore, the basic idea of the NOMAD project is to use a multi-method/multi-parameter approach and to focus on their synergies which allows us to recognize these side effects and therefore suppress them at the same time.

The nuclear industry already applies several statistical methods where the statistical parameters can be calculated by analytical expression. The Charpy impact test itself is an example, where the transition temperature curve is derived from the pool of the measured data by mathematical fitting. In case of multi-parameter, i.e., multi-dimensional nonlinear problems, the required analytical expression cannot be formulated, so other statistical approaches are required, such as machine learning (ML). 

Since the analytical expression that links the input and the output parameters is not known in the case of ML, transparency is essential for the application of ML, especially in safety-critical areas. We paid great attention to providing careful studies of these methods and their validation. We have analyzed separately which NDE parameters (out of the 28) can contribute to the precision or can help the algorithm to recognize and suppress the side effects and which ones are irrelevant and can be left out without any effect. Different types of ML algorithms tested in competition and their performances have been evaluated. Unfortunately, the details of this extensive study are beyond the scope of this paper.

The important outcome of the ML technique is that not only one but several different ML techniques could reach the required precision and reliability, i.e., to keep the DBTT prediction error lower than ±25 °C, which was previously not possible for any of the individual NDE methods independently.

Although many specimens were investigated within the framework of the NOMAD project, the gathered database is extraordinarily limited from the ML point of view. Statistical methods work well on large datasets. This raises a quite frequent problem of ML applications: the insufficient number of the achievable measurement samples or experimental data, i.e., the limited size of the results database. The task of ML methods is to circumscribe and recognize the information that can already be found in the database.

In order to ease this task, different ways of pre-processing the database are studied in order to increase the precision of the classification. The dimension of the database was reduced by eliminating features having no significant importance, i.e., dropping those measurement outputs that cannot contribute to the classification performance. Different methods can be applied for this feature selection, starting with a simple statistical approach (like the Wilcoxon signed-rank test) but also more complex ones like the different ensembles of decision trees. The *k*-fold cross-validation technique can also be used for scoring the different inputs and for optimizing the database. Database cleaning means not only the elimination of the unusable input parameters, but it also covers the possibilities of the data augmentation and imputation. The aim is clear: to provide a better dataset for training of ML algorithms without falsifying or biasing the dataset. However, every step of data manipulation should be transparent, otherwise the technique cannot be accepted in safety-critical areas.

The present results of the individual NDE measurements together with the ML evaluation proved their suitability to characterize the degradation of reactor pressure-vessel steels caused by a simulated operation condition. A calibration/training procedure was carried out on the merged outcome of testing methods with excellent results to predict the transition temperature, yield strength, and mechanical hardness for all of the investigated materials.

The estimation of the radiation damage of a reactor vessel through the cladding is a very challenging problem. Experimental and numerical studies have been carried out to analyze how the base material can be excited through the cladding and how the properties of the cladding and base material, with respect to their changes, due to the irradiation can be separated. These results have already been published in the case of the MAT method [100,101,102].

The results can be useful for the future potential introduction of this (and in general, any) nondestructive evaluation method.

## 11. Summary and Conclusions

The large test program was successfully completed. A total of more than 700 samples were mechanically tested and analyzed. The various steps of the preparation, support, and execution of the multiple hot-cell campaigns allowed the NDE partners to investigate several hundred irradiated samples. As a result, 28 NDE parameters were measured on a variety of materials, specimens, and irradiation conditions. In parallel, the various irradiated specimens were tested destructively to determine the post-irradiation properties, including tensile, Vickers hardness, and Charpy impact transition curves allowing the determination of the DBTT.

Previous to the NOMAD project, none of the NDE methods or their output parameters could reach the precision and reliability that is required for application in determining the DBTT. Irradiation results in complex processes inside the material that affect the recorded quantities in different ways. This is why a standalone NDE method based on a single physical principle has to face the problem of several side effects resulting in the unacceptable scattering of the output parameter. However, as is proven in Appendix B, this large scatter is probably due to the originally existing inhomogeneity of the investigated material. This is a very important conclusion of the project: the possible error of applied magnetic methods is not responsible for the scattering of the measured parameters. 

The basic idea of the NOMAD project is to use a multi-method/multi-parameter approach and to focus on their synergies which allows us to recognize these side effects, therefore suppressing them at the same time.

The nuclear industry already applies several statistical methods where the statistical parameters can be calculated by analytical expression. The Charpy impact test itself is an example, where the transition temperature curve is derived from the pool of the measured data by mathematical fitting. In the case of multi-parameter, i.e., multi-dimensional nonlinear problems, the required analytical expression cannot be formulated, so another statistical approach is required: the machine learning (ML). 

Since the analytical expression that links the input and the output parameters is not known in case of ML, transparency is essential for the application of ML, especially in safety areas. We paid great attention to careful studies of these methods and to their validation. We have analyzed separately which NDE parameters (out of the 28) can contribute to the precision or can help the algorithm to recognize and suppress the side effects and which ones are irrelevant and can be left out without any detrimental effect. Different types of ML algorithms have been tested and evaluated in terms of the competition of their performances. Unfortunately, the details of this extensive study are beyond the scope of this paper.

The important outcome of the ML technique is that not only one but several different ML techniques could reach the required precision and reliability, i.e., to keep the DBTT prediction error lower than ±20 °C, which was not possible at all from previous single NDE methods.

Although hundreds of specimens were investigated within the framework of the NOMAD project, the gathered database is extraordinarily limited from the ML point of view. Thus, we can consider our carried-out work as a kind of feasibility study. The next step should be to extend the dataset. Nevertheless, the present results of the individual micromagnetic measurements together with the ML evaluation proved their suitability to characterize the degradation of reactor pressure-vessel steels caused by the simulated operation condition. A calibration/training procedure was carried out on the merged outcome of testing methods with excellent results to predict the transition temperature, yield strength, and mechanical hardness for all of the investigated materials.

The estimation of the radiation damage of a reactor vessel through the cladding is a very challenging problem. Experimental and numerical studies have been carried out to analyze how the base material can be excited through cladding and how the properties of the cladding and base material, such as their changes due to the irradiation, can be separated. These results have already been published in the case of the MAT method.

Our results, achieved within the NOMAD project, can be useful for the future potential introduction of this (and in general, any) nondestructive evaluation method. 

## Figures and Tables

**Table 1:** Applicability of NDE methods for embrittlement characterization of two kinds of samples.

NDE Method	Charpy Samples	Blocks
Micromagnetic multiparameter microstructure and stress analysis (3MA-X8)	√	√
Piezoelectric ultrasound (Piezo-US)	√	not applicable
Direct current-reversal potential drop (DCRPD)	√	not applicable
Micromagnetic inductive response and Barkhausen emission (MIRBE)	√	√
Magnetic adaptive testing (MAT)	√	√
Thermoelectric power (TEP)	√	√

**Table 2:** Material compositions (wt %).

Material	C	Mn	P	Cr	Mo	Ni	Cu	S	Si
18MND5-W	0.09	1.21	0.018	0.12	0.49	0.96	0.13	0.007–0.011	0.23–0.31
22NiMoCr37	0.2	0.87	0.009	0.39	0.49	0.85	0.06	0.007–0.011	0.23–0.31
A508-B	0.2	1.4	0.01	0.1	0.45	0.74	0.06	0.007–0.011	0.23–0.31
HSST-03	0.25	1.42	0.013	0.48	0.62	0.12	0.12	0.007–0.011	0.23–0.31
A508 Cl.2	0.21	0.75	0.01	0.39	0.6	0.8	0.12	0.007–0.011	0.23–0.31
15kH2NMFA	0.16	0.42	0.012	1.97	0.52	1.29	0.12	0.007–0.011	0.23–0.31

**Table 3:** Results of the Charpy impact tests.

Material	Fast Neutron Fluence [n/cm^2^] × 10^−19^	Irrad. Temp. [°C]	DBTT [°C]	Number of Samples
18MND5-W	0	-	−51	5
	2.88–3.87	150	145	6
	4.57–5.05	260	98	3
	8.57–9.89	260	178	6
22NiMoCr37	0	-	−62	3
	2.99–4.02	260	−6	6
	4.87–6.84	260	29	6
HSST-03	0	-	−6	5
	2.43–2.80	150	161	7
	3.55–4.40	305	34	7
A508-B	0	-	−55	5
	3.80–4.39	150	156	7
	3.28–4.99	305	−5	8
A508 Cl.2	0	-	−33	12
	1.55	100	76	4
	4.38	100	125	4
	7.04	100	126	4
15kH2NMFA	0	-	−51	12
	2.78	100	88	4
	6.83	100	136	4
	7.9	100	124	4

**Table 4:** Results of the Charpy impact tests on specimens cut out from the blocks: cladded blocks top side and bottom side.

	Real Fluence [n/cm^2^] × 10^−19^	Irrad. Temp. [°C]	DBTT [°C]	Number of Samples
**A508 Cl.2** Cladded topside uniform	0	-	−45	3
	2.09	100	90	1
	6.93	100	112	1
	12.70	100	112	1
**A508 Cl.2** Cladded topside attenuated	0	-	−45	3
	1.48	100	94	1
	5.12	100	103	1
	8.86	100	110	1
**A508 Cl.2** Bottom side uniform	0	-	−45	3
	1.72	100	119	1
	6.76	100	145	1
	12.75	100	165	1
**A508 Cl.2** Bottom side attenuated	0	-	−45	3
	4.55	100	133	1
	11.23	100	157	1
	17.40	100	181	1

**Table 5:** Results of the Charpy impact tests on specimens cut out from the blocks: non-cladded blocks.

	Real Fluence [n/cm^2^] × 10^−19^	Irrad. Temp. [°C]	DBTT [°C]	Number of Samples
**A508 Cl.2** Cladded topside uniform	0	-	−45	3
	2.09	100	90	1
	6.93	100	112	1
	12.70	100	112	1
**A508 Cl.2** Cladded topside attenuated	0	-	−45	3
	1.48	100	94	1
	5.12	100	103	1
	8.86	100	110	1
**A508 Cl.2** Bottom side uniform	0	-	−45	3
	1.72	100	119	1
	6.76	100	145	1
	12.75	100	165	1
**A508 Cl.2** Bottom side attenuated	0	-	−45	3
	4.55	100	133	1
	11.23	100	157	1
	17.40	100	181	1

**Table 6:** The 10-fold cross-validation, training, and test scores of the three models [105].

		HLR	SVR	ANN
10-fold cross-validation score	MAE [°C]	13.67	13.58	12.7
Training score	MAE [°C]	9.32	8.68	9.21
	RMSE [°C]	14.49	13.63	12.57
	R^2^	0.97	0.97	0.98
Test score	MAE [°C]	17.1	17.77	16.04
	RMSE [°C]	18.67	20.4	22.08
	R^2^	0.95	0.94	0.93

**Table 7:** A summary of the accuracies of the models. The number of folds *k*, *k*-fold cross-validation scores, training scores, and test scores are reported for each model. The scores are reported as mean absolute errors (MAEs), root-mean-square errors (RMSEs), and R^2^ scores.

Dataset	*k*	*k*-Fold MAE [°C]	$k-\mathrm{Fold}\;R^{2}$	Train RMSE [°C]	Train MAE [°C]	$\mathrm{Train}\;R^{2}$	Test RMSE [°C]	Test MAE [°C]	$\mathrm{Test}\;R^{2}$
Charpy of all RPV steels	10	13.17	-	7.26	6.59	0.99	23.69	15.95	0.92
Blocks: test/train	38	11.58	-	4.99	4.06	1.0	22.17	17.8	0.93
All samples of A508 Cl2	10	13.02	-	8.18	5.79	0.99	17.56	13.47	0.96
Charpy + blocks: 50/50	10	25.68	-	13.1	8.02	0.98	26.21	18.65	0.9

**Figure A1:**
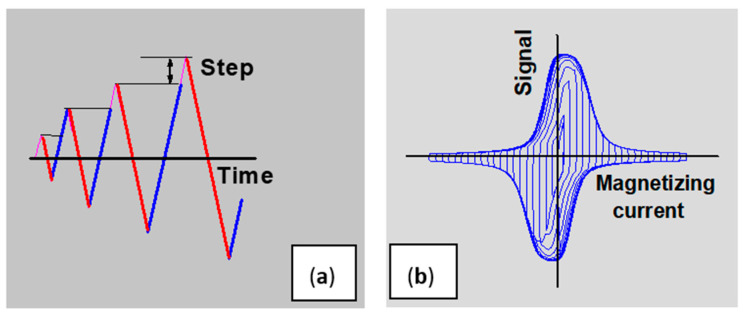
Time variation in magnetizing current (**a**) in the forward (blue) and in the reverse (red) direction and the detected permeability loops (**b**).

**Figure A2:**
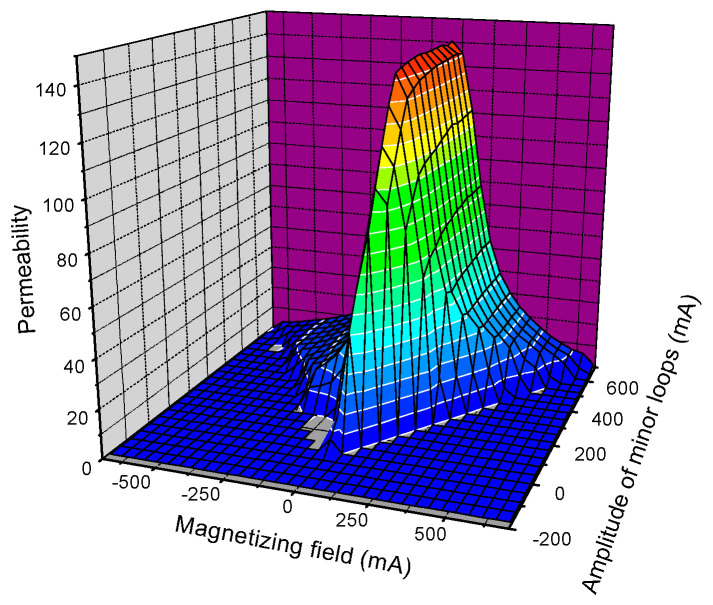
Permeability matrix.

**Figure A3:**
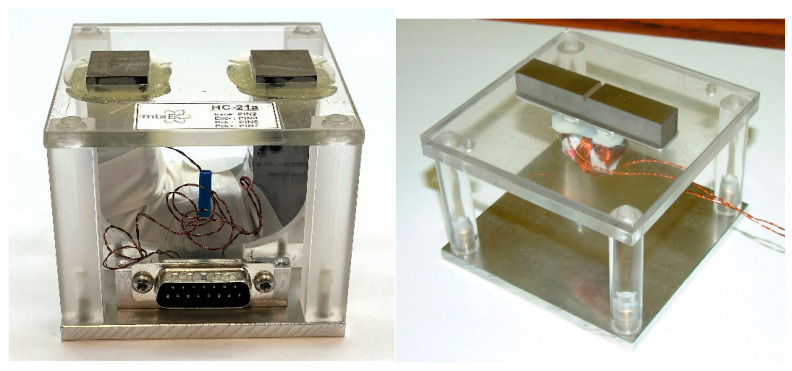
Probes, designed for hot cell MAT measurements. (**Left**) Probe for measuring blocks, (**Right**) probe for measuring Charpy samples [98]. (Here, a sample is also shown, placed on the top (V-notch opposite to the magnetizing yoke.)

**Figure A4:**
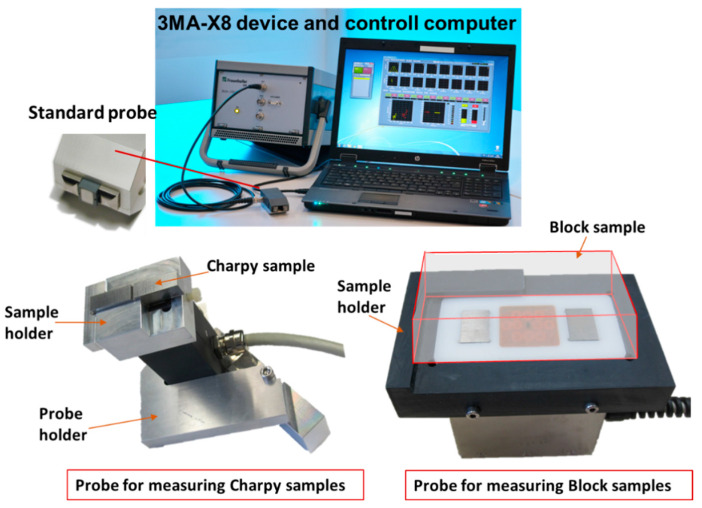
The micromagnetic multiparameter microstructure and stress analysis (3MA)-X8 system, including the 3MA-X8 device, standard probe, and PC (**top**); probe and sample holder for Charpy samples (**bottom left**) and probe and sample holder for block samples (**bottom right**).

**Figure A5:**
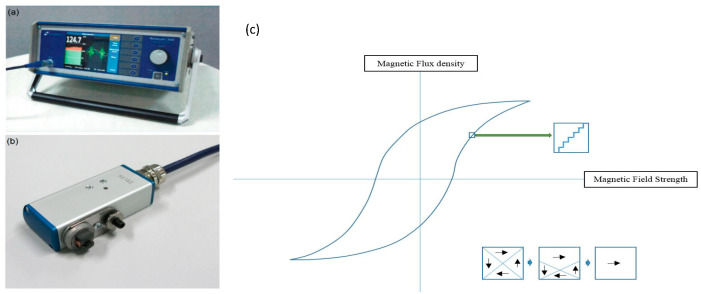
MIRBE setup used in the work: (**a**) noise analyzer and (**b**) sensor; (**c**) Schematic of domain (their orientation are indicated by the arrows) growth during magnetic hysteresis cycle using the MIRBE (BN) method.

**Figure A6:**
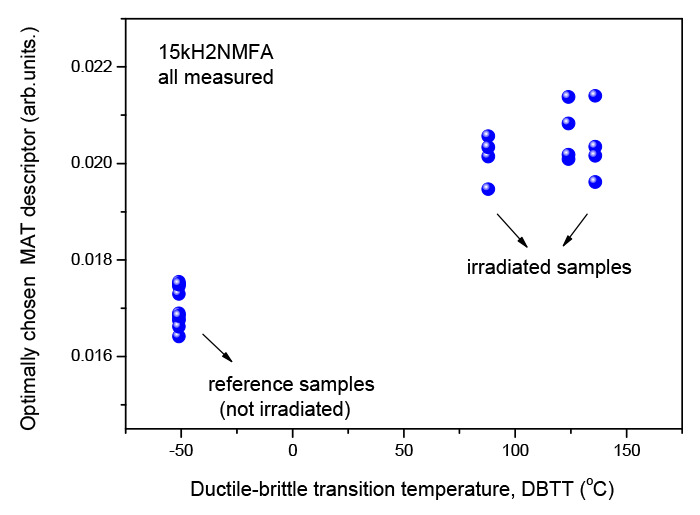
MAT parameter as a function of the DBTT for all measured 15kH2NMFA samples.

**Figure A7:**
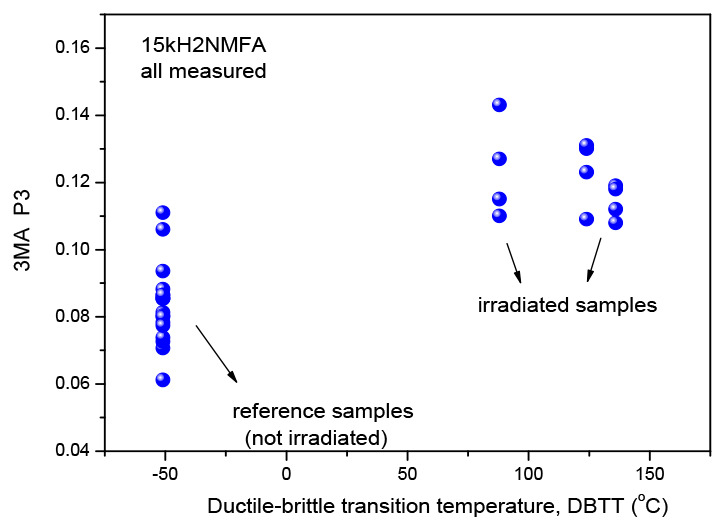
3MA parameter as a function of the DBTT for all measured 15kH2NMFA samples.

**Figure A8:**
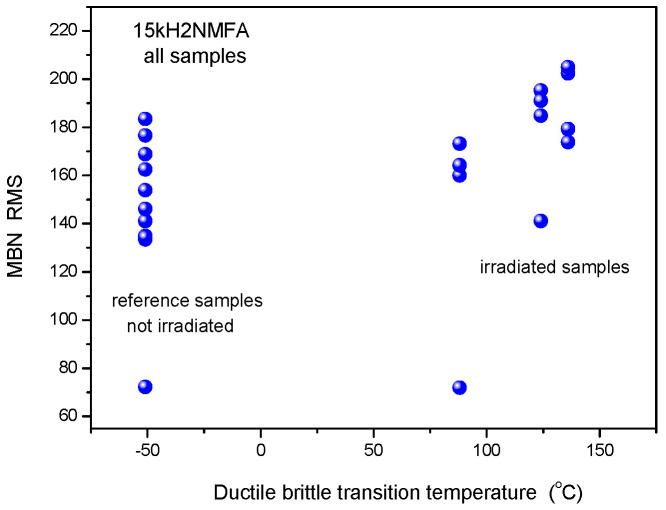
MIRBE parameter as a function of the DBTT for all measured 15kH2NMFA samples.

**Figure A9:**
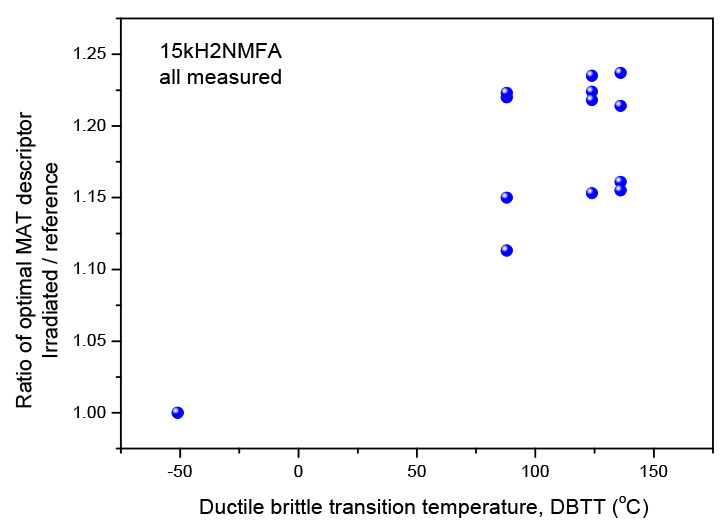
Normalized MAT parameters as function of the DBTT for all 15kH2NMFA samples.

**Figure A10:**
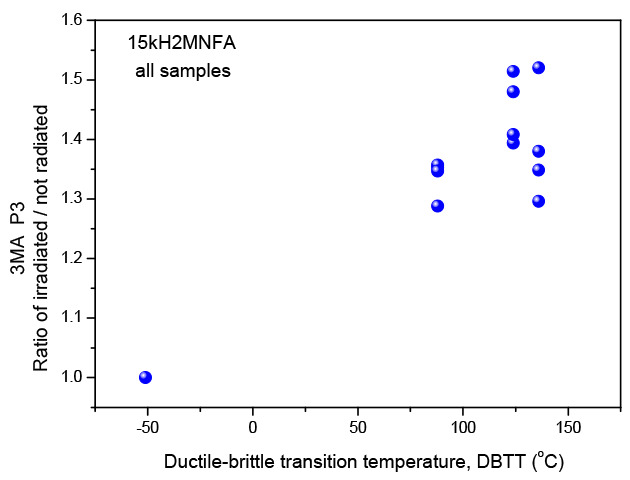
Normalized 3MA parameters as function of the DBTT for all 15kH2NMFA samples.

**Figure A11:**
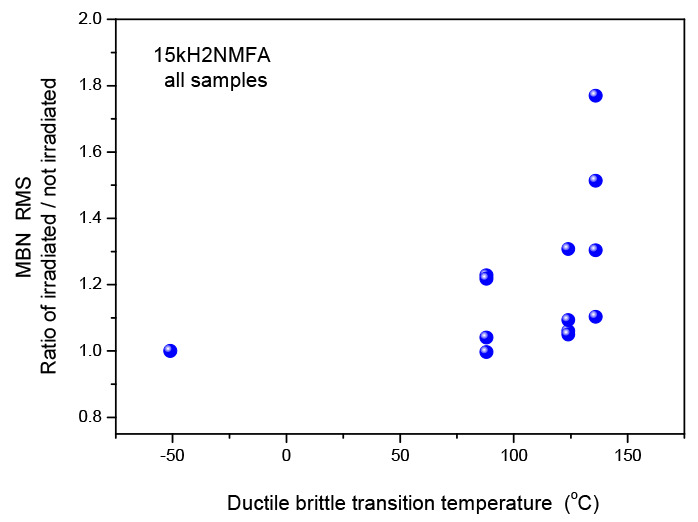
Normalized MIRBE parameters as function of the DBTT for all 15kH2NMFA samples.

**Figure A12:**
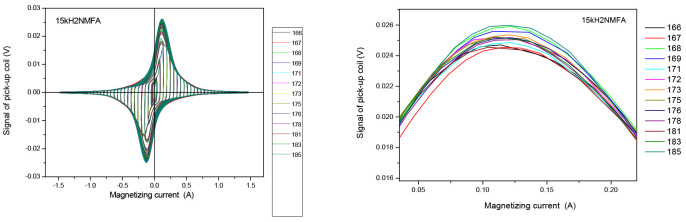
Total permeability loops of 15kH2NMFA samples before irradiation (**left**). The magnified part of the left graph (**right**).

**Figure A13:**
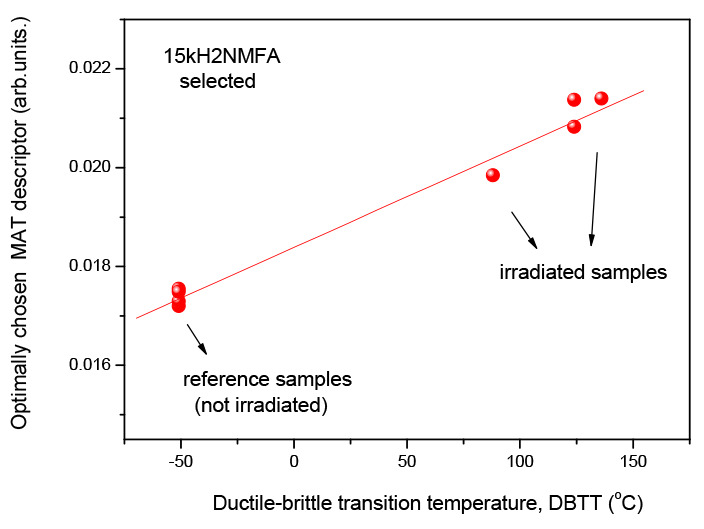
Optimally chosen MAT descriptor as a function of the DBTT for selected 15kH2NMFA samples.

**Figure A14:**
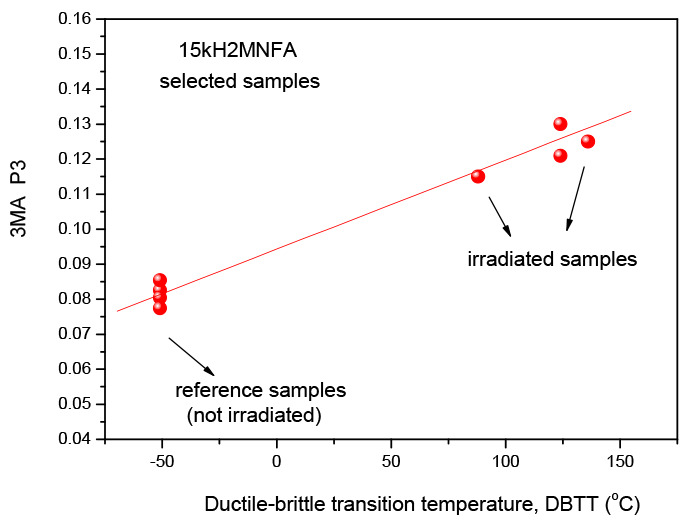
3MA parameter as a function of the transition temperature for selected 15kH2NMFA samples.

**Figure A15:**
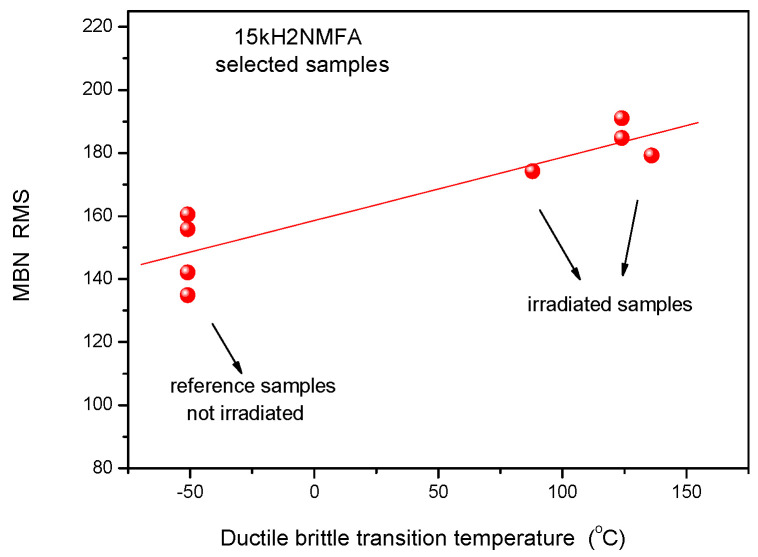
MIRBE parameter as a function of transition temperature for selected 15kH2NMFA samples.

**Figure A16:**
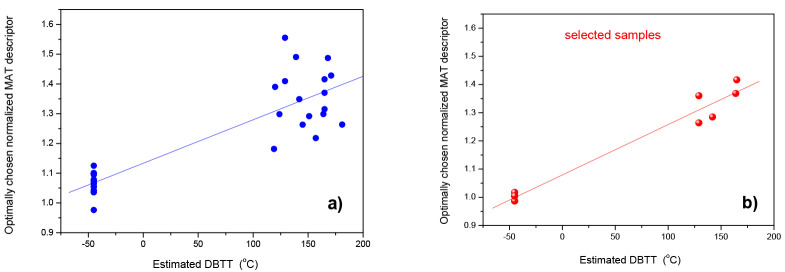
Optimally chosen MAT descriptor as a function of the estimated DBTT on all base material samples (**a**), and the same correlation after magnetic selection (**b**).

## Appendix A. Description of Magnetic NDE Methods

1. MAT

A nondestructive testing method, called magnetic adaptive testing (MAT) was developed for detecting different types of degradation of ferromagnetic materials in the Centre for Energy Research. This method is an expansion of traditional magnetic hysteresis measurements. In contrast to it, where investigated samples are characterized by their single major hysteresis loop, this method studies the complex set of minor magnetic hysteresis loops of each sample within the investigated series. As presented in the theoretical Preisach hysteresis model [111], the complex sets of minor magnetic hysteresis loops contained a lot of information about the material properties. The physical principles of MAT, experimental applications, and method of evaluation of recorded data are described in detail in Ref. [70].

The samples to be measured are magnetized by a magnetizing yoke, which is placed on the surface of the sample. The size of the yoke should be chosen to fit the size of investigated samples. A magnetizing coil and a pick-up are wound on the bows of the yoke. The yoke is a C-shape laminated FeSi transformer core. The current of the magnetizing coil is changed in a triangular-shaped time function by a fixed slope, and the maximum of amplitudes are gradually increased by a pre-defined step in each reverse magnetization cycle. The signal induced in the pick-up coil is proportional to the differential permeability of the measured material.

$$\mu (h_{a},h_{b})=const\times U(h_{a},h_{b})=const\times \partial B(h_{a},h_{bj})/\partial h_{a}\times \partial h_{a}/\partial t$$

where *h_a_*(*t*) is the effective field produced in the magnetizing circuit, and *h_b_* is the amplitude of the minor loops. The time variation in the magnetizing current and the detected permeability loops are shown in Figure A1. Before measurement, the sample is demagnetized by a decreasing amplitude of magnetizing current, so in each case the measurements start on samples having zero magnetic moment.

The detected series of minor permeability loops serve as the base of further evaluation. All sets of hysteresis loops are re-computed into a matrix of elements. These elements are positioned by field coordinates (*h_a_*, *h_b_*). Each matrix element can be used as a magnetic descriptor, which characterizes the structure variation in the investigated sample. As an illustration, the calculated *μ*(*h_a_*, *h_b_*) permeability matrix is presented in Figure A2. If a series of samples are measured, such a matrix is determined for each sample. This matrix contains a lot of information. Other matrices can also be calculated, e.g., the hysteresis matrix by integration of the directly measured permeability values, or the first derivative of the permeability values. Sometimes these matrices characterize the material degradation better than the permeability matrix.

MAT is a comparative method; it does not yield absolute values of magnetic quantities, but it is suitable to follow material changes due to any external effect. For this purpose, another program of evaluation was developed as well: the actual matrix elements are divided by the corresponding matrix elements of the reference sample in its original state. The result is a standardized *μ*(x) matrix, the elements of which contain all of the information about material degradation, and ‘x’ is the so-called independent parameter, which characterizes what happened with the investigated material: plastic or elastic deformation, fatigue, neutron irradiation, thermal treatment, etc.

Degradation functions having different coordinates react differently to the studied degradation. A procedure is used, which is able to pick up those descriptors, which react optimally to the considered mode of degradation of the investigated material. The optimal, the most sensitive, and the most reliable descriptors are used for detecting the structural modifications of the material in question.

The selection of the optimal descriptor increases the reliability and robustness of this method. It is also possible to analyze the known ‘side effects’ of a certain application and to identify those descriptors which are mainly affected by these disturbances. In this way, an opportunity can be established for suppressing measurement uncertainties that can improve the method robustness further.

A careful analysis of potential experimental errors was performed in Ref. [112] and it was found that by taking into account all possible sources of uncertainty, the error of the whole MAT evaluation is less than 1%.

2. 3MA-X8

Micromagnetic techniques are widely used for the nondestructive characterization of the material properties of ferromagnetic steels. They are based on the correlation between the magnetic properties of ferromagnetic materials and their mechanical–technological characteristics, which are dependent on the microstructure. This correlation is related to the microstructure interaction with both the magnetic structure (domain (Bloch) walls), as well as the dislocations [63,64,65]. Fraunhofer IZFP has developed the 3MA technique (micromagnetic multiparameter microstructure and stress analysis), which indirectly and nondestructively determines mechanical material properties using a one-sided access probe. The 3MA technique is based on a combination of several magnetic methods and has been described in previous studies in detail [63,64,65]. The latest implementation of 3MA is the 3MA-X8 principle, which has been applied in this work. The 3MA-X8 probe records parameters derived from three micromagnetic methods that rely on a single sensor coil wound on a U-shaped core. The 3MA-X8 probe contains an electromagnet that excites a magnetic field of two superimposed frequencies in the sample. The time domain signals of drive voltage and drive current depend on the properties of the sample contacted by the probe. These signals are analyzed in order to extract up to 21 characteristic features that describe the magnetization behavior and then can be correlated with the mechanical material properties.

The following three micromagnetic methods are used in 3MA-X8: eddy current impedance analysis, incremental permeability analysis, and harmonics analysis. They differ in terms of the interaction depth and mechanisms and deliver features that qualitatively correlate with material properties. Figure A4 shows the 3MA-X8 device including the used probes and a PC. The 3MA-X8 device is controlled by the modular measuring system (MMS) software. For Charpy samples, a standard probe was used, and for measuring the block shaped samples, a large adapted probe was designed and built up. Sample/probe holders were used to improve reproducibility by minimizing the positioning variations.

3. MIRBE

The micromagnetic inductive response and Barkhausen emission (MIRBE) technique is a nondestructive examination technique for microstructural changes, the detection of surface defects, and case hardening levels [50,60]. In more depth, the method works where magnetic changes over time along with the Barkhausen noise analysis provide an insight into the material characteristics/properties. MIRBE has its origins in the B-H hysteresis loop which is not a smooth curve as the magnetic flux density versus the intensity of the magnetic field results in a curve that is instead described as a nonlinear step function. These steps correlate with the irregular fluctuations in the magnetization when energized from the cyclic excitation provided by ferrous yokes to excite the material area under test. These steps or jumps of domains forming Barkhausen noise (BN) are provided from the magnetic domain motion which is the basis of the Barkhausen signal. Moreover, until the applied field is increased sufficiently, pinning sites tend to restrict the moving domain wall. When the applied field is reached, sudden changes in magnetization result which are produced by the sudden and discontinuous movement of the domain walls. In the case of microstructural characteristics, “defects” such as dislocations, precipitations, and segregations which cause the pinning of the moving domain walls promote Barkhausen signal changes. Barkhausen noise is measured from a receiving ‘pick-up coil (independent to the energizing yoke)’ in the form of a voltage signal of significant surface eddy currents experienced near the surface of the material.

BN measurements were undertaken using a magnetic Barkhausen noise analyzer (Rollscan 350) with a general-purpose sensor developed by Stresstech [113]. The magneto elastic parameter (mp) signifying the RMS (root mean square) value is expressed as a function of the magnetizing voltage, current, and frequency. Each measurement consisted of periodic bursts of BN signals for a set duration of ten seconds, and the BN RMS is calculated from such signal bursts. The RMS of the MBN signals is given as

$$\mathrm{RMS}=\sqrt{\frac{{\sum_{i=1}^{n}yi}^{2}}{n}}$$

where *n* is the total number of BN signals captured in the particular frequency range, and *y_i_* is the amplitude of the individual burst.

The main instrumentation input parameters are voltage and frequency, and these are determined from voltage and frequency sweeps giving an optimum ‘trade-off’ value for a specific material under test. Both sinusoidal and triangular waveforms can be used to energize the surface; due to saturation issues with the triangular waveform, the sinusoidal waveform was used. It should also be noted that the frequency of the applied field affects the depth at which the Barkhausen noise reading is obtained. The frequency is inversely proportional to the depth of penetration. A penetration depth between 0.01 mm and 1 mm was achieved when a band pass filter of between 70 kHz and 200 kHz was selected for channeling the pick-up signals of interest.

The scatter of the magnetic output responses vs. the DBTT was also found with Barkhausen noise. It was considered that such a scatter is due to the material microstructure differences, as well surface quality in terms of surface roughness. Measurement uncertainty, however, was minimized as much as possible; this is in terms of sensor pick-off, surface quality, and applied force. The measurement testing regime used a three times sensor pick-off (physical movement of the sensor to and from the same position maintained) followed by five measurements each time the sensor touched the surface of the material. The MIRBE setup used for the measurements can be seen in Figure A5.

## Appendix B. Interpretation of the Scatter of Points

The results of different nondestructive measurements are presented in Section 8. As a general conclusion, a reasonable correlation was found between the nondestructively measured magnetic parameters and the destructively measured transition temperature, regardless on the type of measurement (MAT, 3MA, MIRBE). However, the measurement points significantly scatter in all of these experiments. It has been supposed that the reason for this scatter can be the local material inhomogeneity [112]. It is also known that the embrittlement also depends on the initial material conditions, in spite of the fact that the investigated samples were cut from the same block. Considering the importance of these nondestructive measurements on RPV steel for the potential future inspection of nuclear reactors, the results should be analyzed and verified carefully. This was the purpose of the work, described in Ref. [114], and in this section the most important results and conclusions are summarized. The results of the measurements presented in Section 8 are also considered here, but these results are evaluated from a different point of view. In Ref. [114], the measurement results of two series of Charpy specimens (made of two different types of RPV steel) were analyzed. Measurements were performed both before and after neutron irradiation, and the abovementioned three magnetic methods were applied on the same specimens. In this section, only the measurements made on the 15kH2NMFA material are presented, but very similar results were also obtained on the A508 Cl.2 material.

The same procedure was taken for a series of blocks; this result is also presented below.

1. Results of MAT, 3MA, and MIRBE measurements made on all Charpy samples
  (1) Evaluation of data without normalization

It is shown in Figure A6 how the optimally chosen MAT descriptor depends on the DBTT. A similar result with the 3MA measurement is shown in Figure A7, while the result of MIRBE measurements can be seen in Figure A8. These results are identical to the results presented in Section 8.

It is evident that neutron irradiation caused a clearly measurable modification of the magnetic parameters. A more or less linear correlation seems to exist between the magnetic quantities and the DBTT. However, the most visible effect is the large scatter of the points, regardless of the actual method of measurement.

On the other side, it is also evident in the above figures that even the magnetic parameters of the reference (not irradiated) samples scatter a lot. This experience gives a possible explanation of the scatter of the points: the samples themselves behave differently, in spite of the fact that they were cut from the same block. Only the magnetic measurements reflect this material inhomogeneity. Consequently, it is not surprising that the points also scatter after irradiation. To obtain an impression of the behavior of individual samples, it will be investigated in the next subsection how the magnetic behavior of individual samples is modified due to neutron irradiation.

(2) Evaluation of normalized data

In Figure A6, Figure A7 and Figure A8, the measurement results are given without marking the individual samples. Perhaps a more useful method of presentation is to consider the modification of magnetic parameters for each individual sample. Because of this, other graphs are shown in Figure A9, Figure A10 and Figure A11. In these figures, the changes in magnetic parameters are given with respect to the same magnetic parameter measured on the same sample before irradiation. It is for this reason that the first point (Ratio = 1) is the same for all samples, and the other points are connected with individual samples. These points show how the magnetic parameters of a given sample are modified due to irradiation.

It can be clearly seen in the above figures that the scatter of the points is also rather large in the normalized cases. This can be considered as direct proof that the scatter of the points is the result of the originally different behavior. The neutron irradiation generates different material embrittlement, depending on the individual properties of the samples.

2. Selection of samples

The influence of neutron irradiation is investigated in the above subsections for the case where all measured samples are taken into account. But it is very clearly seen that even the reference samples are very different from the point of view of magnetic properties. It is not surprising that they also behave differently after irradiation. In this subsection, it is shown how a magnetic selection of samples can be done. This selection is based on permeability measurements of the samples. In the next subsection, it will be shown how the correlation between the magnetic parameters and the DBTT looks if only the selected samples are taken into account. It is important to emphasize that this selection was made before any further magnetic measurement and evaluation of the irradiated samples.

The permeability loops were measured on the reference samples before irradiation. This means that the selection does not take into account the material embrittlement generated by the neutron irradiation. It simply reflects the behavior of the samples in initial conditions.

No backward reasoning has been used to decide which measured points fit best to our hypothesis. To clarify this statement, the details of this selection process are briefly given:

- A large scatter of all magnetic parameters measured on both the reference and irradiated samples was observed;
- Regardless of the result of the magnetic measurements, we compared the magnetic behavior of the reference samples with each other. It was found that four samples behaved similarly from the magnetic point of view;
- The MAT, 3MA, and MBN evaluations (not measurements!) were repeated, taking into account only those selected samples. It is evident that this selection cannot give any information about the behavior of the irradiated samples, considering that it is conducted prior to the irradiation.

The series of permeability loops with increasing magnetization detected on the 15kH2NMFA samples can be seen on the left side of Figure A12. On the right side of the figure, the magnified parts of the loops are shown. For the sake of better visibility, here the envelope of the large amplitude minor loops is shown. The difference between loops can be clearly seen, and an easy selection can be made. Four samples behave similarly: Nos. 172, 173, 178, 183.

3. Results of 3MA, MAT, and MBN measurements considering selected samples only

In this subsection, it is shown how the scatter of the points looks if the evaluation of magnetic parameters has been performed by taking into account only the magnetically pre-selected samples. The results can be seen in Figure A13, Figure A14, and Figure A15, respectively.

4. Results of MAT measurements made on blocks

In this subsection, the results of the same procedure as described above are given, but now those measurements are considered which were performed on A508 Cl.2 base metal blocks before and after neutron irradiation [102]. Again, the measurement points are the same as those presented in Section 8 but after performing the magnetic selection, and only MAT results are considered. The correlation between the MAT parameters and the DBTT is shown in Figure A16, before and after selection.

5. Discussion

The above described analysis revealed that the large scatter experienced is most probably connected with the different behavior of the samples. The measurement errors of the applied magnetic methods are not responsible for this scatter. In the case of MAT, a careful analysis of potential experimental errors was performed [112] and it was found that by taking into account all of the possible sources of uncertainty, the error of the whole MAT evaluation was less than 1%.

By comparing Figure A13, Figure A14 and Figure A15 with either Figure A6, Figure A7 and Figure A8 or with Figure A9, Figure A10 and Figure A11, it is clearly seen that the scatter of points significantly decreased if the evaluation of the measurement results was performed only on the selected Charpy samples. Obviously, a linear correlation with a low scatter of points was found between the magnetic parameters and the DBTT. This statement is supported by the fact that all the three applied methods resulted in the same outcomes: Neither the correlation between the magnetic quantities and the transition temperature nor the scatter depend on the actual method of measurement. This fact is very promising for the future practical application of magnetic methods. The results of different methods therefore verify each other.

Furthermore, the influence of proper magnetic selection is identical for both Charpy and block samples. This fact makes evident that this phenomenon also does not depend on the size and shape of the investigated specimens.

6. Conclusions

It was clearly demonstrated by our experiments that the neutron-irradiation-generated embrittlement is very well characterized with the low scatter of the measured magnetic parameters if the parameters of the non-irradiated samples are close to each other. The selection can be easily performed by magnetic measurements.

It can be stated—based on the above experimental results—that magnetic parameters seem to better characterize the neutron-irradiation-induced material embrittlement than the traditionally used destructive methods, e.g., transition temperature. This is the most important conclusion of this work. The scatter of the magnetic parameters is significantly lower than the scatter of destructive Charpy impact tests. Furthermore, the magnetic measurements characterize the individual samples, contrary to the DBTT values determined by the Charpy method which provides only statistical values on the sample sets.

Another important conclusion was also found in that the local material inhomogeneity strongly influenced the neutron-irradiation-generated material embrittlement. Different parts of RPV, even if they are cut from the same block, are hardened differently. Local material conditions are responsible for the different embrittlement of the material caused by the same dosage of neutron irradiation.

These results mean a serious argument for the application of magnetic measurements in the reactor industry and in the very near future.
